# RUVBL2 Regulates Microglia Metabolic Reprogramming to Mediate Stress Granules Aggregation Exacerbating Postoperative Delirium in Aged Mild Cognitive Impairment Rats

**DOI:** 10.1111/acel.70458

**Published:** 2026-04-03

**Authors:** Lin Zhang, Zixuan Wang, Chenyi Yang, Xinyi Wang, Xing Liu, Haonan Zhang, Huan Liu, Huihui Liao, Jun Chen, Haiyun Wang

**Affiliations:** ^1^ The Third Central Clinical College of Tianjin Medical University Tianjin China; ^2^ Department of Anesthesiology Tianjin University Central Hospital Tianjin China; ^3^ Department of Anesthesiology Central Hospital, Tianjin University Tianjin China; ^4^ Tianjin Key Laboratory of Extracorporeal Life Support for Critical Diseases Tianjin China; ^5^ Tianjin University Tianjin China

**Keywords:** metabolic reprogramming, microglia, neuroinflammation, postoperative delirium (POD), RUVBL2, stress granules (SGs)

## Abstract

Postoperative delirium (POD) accelerates the transition from mild cognitive impairment (MCI) to Alzheimer's disease (AD) in elderly patients. Microglial metabolic reprogramming, a pivotal aspect of the immune‐inflammatory response, modulates microglia–neuron interactions and postoperative cognitive function through microenvironmental alterations. Aberrant overexpression of RUVBL2 disrupts metabolic homeostasis, leading to stress granule (SG) aggregation and fibrosis. This study investigated the role of RUVBL2 in regulating metabolic reprogramming to mediate SG formation, with the aim of identifying novel prognostic targets for inhibiting glycolysis and mitigating POD‐induced MCI progression. A POD model was established in aged MCI rats using 3% sevoflurane anesthesia for 3 h, combined with open reduction and internal fixation (ORIF). Multimodal magnetic resonance imaging (MRI) was employed to assess postoperative cognitive function. Glycolytic and oxidative phosphorylation (OXPHOS) activities in primary hippocampal microglia were quantified by extracellular acidification rate (ECAR) and oxygen consumption rate (OCR). Lentiviral‐mediated RUVBL2 expression modulation was performed to verify its role in microglial metabolic reprogramming. Postoperative hippocampal microglia underwent metabolic reprogramming from OXPHOS to glycolysis, with RUVBL2 expression correlating positively with POD progression. Elevated RUVBL2 expression drove metabolic reprogramming, while RUVBL2 knockdown inhibited this process, alleviated pro‐inflammatory microglia‐induced neuroinflammation and SG aggregation, and improved spontaneous neural activity and hippocampus‐dependent cognitive deficits. In primary hippocampal microglia, RUVBL2 knockdown enhanced OXPHOS‐related OCR and reduced glycolysis‐associated ECAR, producing a synergistic neuroprotective effect. These findings reveal the critical role of RUVBL2 in regulating POD, highlight metabolic reprogramming as a novel therapeutic target, and suggest RUVBL2 as a promising intervention strategy for POD.

## Graphical abstract

RUVBL2 mediates stress granule aggregation by regulating microglial metabolic reprogramming, thereby exacerbating postoperative delirium in aged rats with mild cognitive impairment.

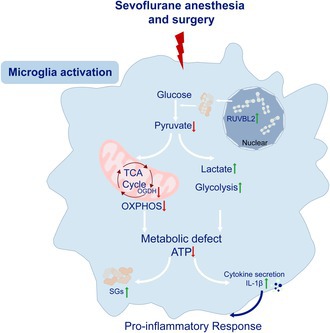

**Abbreviations**
ALFF: amplitude of low‐frequency fluctuation
Arg1: arginase 1
BOLD‐fMRI: blood oxygenation level dependent magnetic resonance imaging
CCAs: common carotid arteries
CNS: central nervous system
DII: DNA‐binding domain II
ECAR: extracellular acidification rate
ELISA: enzyme‐linked immunosorbent assay
G3BP1: GTPase‐activating protein (SH3 domain) binding protein 1
IBA1: ionized calcium‐binding adapter molecule 1
IL‐10: interleukin‐10
IL‐1β: interleukin‐1 beta
LLPS: liquid–liquid phase separation
LPS: lipopolysaccharide
MCI: mild cognitive impairment
MWM: morris water maze
NO: nitric oxide
NOR: novel object recognition
Nos2: nitric oxide synthase 2
OCR: oxygen consumption rate
ORIF: open reduction and internal fixation
OXPHOS: oxidative phosphorylation
PND: perioperative neurocognitive disorders
POD: postoperative delirium
SG, SGs: stress granules
TCA: tricarboxylic acid cycle
TIA1: T‐cell intracellular antigen 1

## 1 Introduction

Postoperative delirium (POD) is the most prevalent neurological complication in elderly patients following surgery, with an overall incidence of approximately 23%–28% (Evered et al. 2021; Wildes et al. 2019). In high‐risk major surgeries, the incidence may increase to 45%–56% (Adelaars et al. 2025; Igwe et al. 2023). POD not only extends hospitalization but also significantly elevates overall mortality (2.8–4 times) (Lander et al. 2025). Research suggests that POD induces persistent cognitive dysfunction and accelerates Alzheimer's disease (AD) progression in patients with pre‐existing mild cognitive impairment (MCI) (Goldberg et al. 2020; Popp 2013; Swarbrick and Partridge 2022). Approximately 63% of patients with preoperative cognitive impairment develop AD within 3 years following POD (Olofsson et al. 2018). However, POD remains a clinical syndrome characterized by poorly understood pathophysiology, underdiagnosis, and unclear mechanisms. To date, no specific preventive measures for POD have been identified, and preventing the accelerated progression from MCI to AD due to POD remains a critical and unresolved challenge.

Microglia, the primary innate immune cells in the brain, play a central role in monitoring the microenvironment (Jung and Mook‐Jung 2020). However, their aberrant activation and dysfunction can trigger neuroinflammatory responses, contributing to neurotoxicity and becoming a key factor in POD (Ishii et al. 2025; Liu et al. 2021). Recent studies indicate that activation of the pro‐inflammatory phenotype in microglia involves dynamic metabolic reprogramming and a pronounced enhancement of glycolytic pathways, which play a pivotal role in POD (Guillot‐Sestier et al. 2021). This metabolic transition not only reflects shifts in cellular energy metabolism but is also closely tied to immune‐inflammatory responses during microglial activation (Fang et al. 2025). In mouse models of AD (e.g., 5XFAD and APP/PS1 mice), microglia display a hyperglycolytic state around Aβ plaques, exacerbating neuroinflammation. This state is associated with reduced phagocytosis of Aβ and impaired migratory activity, accelerating tau pathology propagation (Holland et al. 2018; McIntosh et al. 2019; Pan et al. 2022). A positive feedback loop of glycolysis, lactate accumulation, and enhanced glycolysis further exacerbates microglial dysfunction.

When cells are exposed to external stressors such as oxidative stress, temperature changes, or viral/toxin infections, they initiate integrated stress responses that lead to the formation and assembly of stress granules (SGs) (Costa‐Mattioli and Walter 2020). While the formation of SGs is a protective mechanism to maintain cellular translation under stress, their irreversible aggregation due to abnormal phase transitions contributes to neurotoxicity and serves as a key factor in neurodegenerative diseases (Cui et al. 2024; Li et al. 2022). ATP depletion impedes SG clearance (Wang et al. 2022). Our previous study found that knockdown of RUVBL2, a member of the AAA^+^ protein superfamily, which is ubiquitously expressed and highly conserved in the brain, elevated postoperative ATP levels in aged MCI rats. This elevation accelerated the disassembly of neuronal SGs and mitigated neurocognitive dysfunction (Wang, Yang, et al. 2025). In response to pathological triggers such as inflammatory factors or protein aggregates, microglia become dysfunctional, altering communication with neurons, exhibiting aberrant chemotaxis, and displaying reduced phagocytic activity. This dysfunction leads to the accumulation of misfolded proteins and soluble oligomers, particularly amyloid β (Aβ), triggering neuronal apoptosis and subsequent nerve damage (Depp et al. 2023; Marinelli et al. 2019). Although RUVBL2 plays a critical role in regulating energy metabolism and SG dynamics in neurons, its regulatory mechanisms in microglia remain largely unexplored, offering valuable insights into microglia–neuron interactions.

Whether RUVBL2 influences the progression of POD in aged MCI rats by modulating microglial metabolic reprogramming and subsequently inducing pro‐inflammatory responses and SG aggregation has not been studied. Therefore, this research aims to investigate whether RUVBL2 serves as a key regulator of microglial metabolic reprogramming, contributing to the pathogenesis of POD.

## 2 Materials and Methods

### 2.1 Animals

Specific pathogen‐free (SPF) healthy male Sprague–Dawley (SD) rats (aged 8–9 months, weight 450–550 g) and neonatal SD rats (24 h old) were supplied by Beijing Vital River Laboratory Animal Technology Co. Ltd. All rats were housed in SPF‐grade animal housing rooms, with ambient temperature and humidity maintained within an optimal range. A 12‐h light/dark cycle was maintained, and the rats were provided with standard chow and clean drinking water, allowing ad libitum access to food and water. All experimental protocols received approval from the Institutional Animal Care and Use Committee of Nankai Animal Resource Center, Nankai University (approval number: 2023‐SYDWLL‐000623). The animal husbandry and experimental protocols complied with the principles of laboratory animal care (NIH publication No. 86–23, revised 1985).

### 2.2 Establishment of the Aged MCI Rat Model

The rats were housed in a safe and quiet environment until they reached 24 months of age (weight 650–750 g); rats were subjected to bilateral common carotid artery (CCA) stenosis according to the protocol of our previous study, resulting in severe narrowing and establishing an aged MCI model (Wang et al. 2020). Fasting was implemented for 12 h prior to surgery. Anesthesia was induced via intraperitoneal injection of 50 mg/kg pentobarbital sodium (China National Pharmaceutical Group Corp. Ltd., Shanghai, China), and adequate anesthesia was confirmed upon complete loss of the rat's righting reflex, with spontaneous breathing maintained during the operation. Neck hair was removed, and the skin was disinfected and draped. A 1.5 cm midline vertical incision was made on the neck, subcutaneous tissues and muscles were separated layer by layer, and the bilateral CCA were bluntly exposed with careful protection of the accompanying vagus nerves. A flattened syringe needle (diameter ≈0.5 mm) was inserted 1.5 cm proximal to the carotid bifurcation (junction of internal and external carotid arteries) and ligated together with the CCA. The needle was then removed, and the wound was flushed and sutured. All procedures were conducted under aseptic conditions, with a heating pad maintaining the rat's body temperature at 36.5°C–37.5°C. Sham‐operated rats underwent similar procedures (CCA isolation via blunt dissection) without ligation‐induced stenosis. Postoperatively, penicillin (400,000 IU; Hapharm Group Co. Ltd., Heilongjiang, China) was administered routinely to prevent infection.

### 2.3 Lentivirus Construction

Lentiviruses for RUVBL2 overexpression (LV‐RUVBL2), RUVBL2 knockdown (LV‐shRNA‐RUVBL2), and control empty vector (LV‐Scramble) were obtained from Brain Case Co. Ltd. (Brain Case Co. Ltd., Shenzhen, China). We designed three shRNA sequences targeting RUVBL2 in rats. After evaluation, the shRNA with the highest knockdown efficiency was selected for the experiment. The forward sequence is: 5′‐GGA GGA GAC AGA GAT CAT TGA TTC AAG AGA TCA ATG ATG ATC TCT GTC TCC TCC‐3′ (Wang, Yang, et al. 2025).

### 2.4 Stereotactic Intracerebral Injection

Male aged MCI rats (body weight: 650–750 g) were fasted for 12 h prior to the experiment. Anesthesia was induced and maintained using isoflurane inhalation. Once adequate anesthesia was confirmed, the rats were fixed onto a Model SA‐150 stereotaxic injector (Shanghai Yuyan Instrument Co. Ltd., Shanghai, China). Target coordinates were determined based on the standard rat brain stereotaxic atlas. Two injection sites were selected bilaterally within the hippocampal tissue of each rat. Craniotomy was performed at each site, followed by microinjection at the corresponding target loci. The specific stereotaxic coordinates are as follows: Site 1: Anteroposterior (AP): −1.7 mm, Mediolateral (ML): ±1.1 mm, Dorsoventral (DV): −2 mm from the skull surface; Site 2: AP: −2.9 mm, ML: ±3 mm, DV: −2.7 mm. A 5 μL Hamilton syringe (Hamilton Company, Reno, NV, USA) was preloaded with 2 μL of LV‐Scramble, LV‐shRNA‐RUVBL2, or LV‐RUVBL2 lentivirus (1 × 10^8^ transduction units [TU]/mL) for stereotaxic injection into the rat hippocampus at a rate of 0.1 μL/min. The needle was left in place for 10 min to allow for adequate diffusion within the brain tissue, then slowly withdrawn. The surgical site was disinfected, cleaned, and sutured. Postoperatively, penicillin (400,000 IU; Hapharm Group Co. Ltd., Heilongjiang, China) was administered to prevent infection.

### 2.5 Open Reduction and Internal Fixation (ORIF) Surgery and Experimental Grouping

To simulate clinical anesthesia and surgical trauma, the Sevo, LV‐Scramble, LV‐shRNA‐RUVBL2, and LV‐RUVBL2 groups were exposed to 3% sevoflurane (Marushi Pharmaceutical Co. Ltd., Osaka, Japan) for 3 h and underwent ORIF for tibial plateau fractures. Aged MCI rats were fasted for 12 h before anesthesia and surgery. The rats were secured on an operating table equipped with a thermostatic blanket to maintain stable core body temperature during the procedure. All surgical operations followed strict aseptic techniques: routine hair removal was performed at the surgical site on the left hind paw, followed by disinfection with povidone‐iodine and sterile draping. The skin and subcutaneous tissue were incised layer by layer, and muscle tissue and periosteum were bluntly dissected to fully expose the midshaft of the tibia. A transverse fracture model at the tibial midshaft was created using surgical hemostats, followed by insertion of a 0.3 mm intramedullary nail for internal fixation. After re‐disinfecting the surgical site, the subcutaneous tissue and skin were sutured layer by layer to close the incision. The Sham group received only local infiltration anesthesia with 2% lidocaine, where the tibial region was exposed and immediately sutured without fracture induction or fixation. A heating pad was used intra‐ and postoperatively to maintain normal body temperature, and penicillin (400,000 IU) was administered to prevent infection.

Aged MCI rats were randomly assigned to the following groups (*n* = 10 per group, Table 1).

**TABLE 1:** Experimental grouping of animals.

Experimental grouping	Group name	Model establishment
Group 1	Control	MCI
Group 2	Sham	MCI +2% Lidocaine local anesthesia
Group 3	Sevo	MCI + 3% Sevoflurane (3 h) + ORIF
Group 4	LV‐Scramble	MCI + 3% Sevoflurane (3 h) + ORIF + empty vector LV‐Scramble
Group 5	LV‐shRNA‐ RUVBL2	MCI + 3% Sevoflurane (3 h) + ORIF + LV‐shRNA‐RUVBL2 knockdown
Group 6	LV‐ RUVBL2	MCI + 3% Sevoflurane (3 h) + ORIF + LV‐RUVBL2 overexpression

### 2.6 Morris Water Maze Test

The Morris water maze test (MWM) was performed 30 days after surgery to narrow the bilateral common carotid arteries to screen for successful modeling of MCI rats. A black opaque cylindrical water maze was filled with water, and a circular platform was placed approximately 2 cm below the water surface in the third quadrant of the maze. The time from the time the rat entered the water until it found and climbed onto the platform was recorded as the escape latency. On Day 1, rats were allowed to explore the water freely for 1 min to adapt to the environment; on Days 2–5, rats were trained by placing them into the water from each of the four quadrants four times a day; on Day 6, the platform was removed, and rats were placed into the water from the first quadrant, and the time when the rats crossed the platform for the first time was recorded as the avoidance latency period. The escape latency of the unmodeled rats was set as the reference value. If the escape latency of the model rats was < 20% compared with that of the unmodeled rats, then the MCI modeling was successful (Wang et al. 2020).

### 2.7 Barnes Maze Test

The Barnes maze consists of a black opaque circular platform with 18 equally spaced circular holes around the perimeter. A square safety box (target box) is placed beneath one of the holes, designated as the target hole. Six days prior to the ORIF surgery, the rats were acclimated to the maze environment and the target hole to reduce stress from the novel setting. This was followed by a 5‐day training period. Training was conducted once daily per rat starting at 10:00 AM, with a 5‐min observation period. In a dark room, the rat was first placed at the center of the platform and confined in an opaque box for 30 s to allow it to calm. Following this, the light was turned on, the box was removed, and the timer was started immediately. An escape was considered successful when the rat's body fully entered the target box and remained there for 5 s. The light was then turned off to indicate the safety of the target box. If the rat failed to enter the target box within 5 min, the trial was deemed unsuccessful: the rat was placed into the target box, the light was turned off, and after a 30‐s acclimation period, the rat was returned to its home cage. Following each trial, the platform and target box were cleaned with 75% ethanol to prevent interference from olfactory cues. Testing commenced 3 days after ORIF surgery. The target box was removed from beneath the target hole, and the maze platform was confirmed to be free of residual odors. The experimental environment was kept consistent with the training period, and the rat was allowed to explore freely for 5 min. The number of visits to the target hole and the latency to reach the target hole were recorded.

### 2.8 Novel Object Recognition Test

The novel object recognition (NOR) test was performed 2–3 days after ORIF. On the 2nd postoperative day, rats were first placed in a black box without objects for 5 min of acclimation, without intervention to eliminate abnormal stress responses. Subsequently, two identical objects (A1, A2) were placed at diagonal positions in the box for a 5‐min training session. Two hours later, object A2 was replaced with a novel object B for a 5‐min test; 24 h later, object B was replaced with another novel object C for an additional 5‐min test. After each exploration session, the box was cleaned with 75% ethanol to eliminate residual odors. The NOR index was calculated as the ratio of the time spent exploring the novel object to the total exploration time.

### 2.9 Magnetic Resonance Imaging

Aged MCI rats underwent functional brain imaging on Day 3 after ORIF with multimodal scanning using a Bruker 9.4T BioSpec 94/30 system (Bruker, Ettlingen, Germany). Inhalation anesthesia of 1.5%–2% isoflurane was maintained throughout the experiment, and the core body temperature was maintained at 37.0°C ± 0.5°C by a water‐circulating temperature control system, with simultaneous monitoring of heart rate, respiratory rate (60–70 breaths/min), and paw oxygen saturation. Head fixation was realized by using double ear bars combined with occluder. Frequency tuning and homogeneous field correction were completed, and whole brain T2WI (repetition time (TR)/echo time (TE) = 2500/33 ms, layer thickness = 1 mm, matrix = 256 × 256, field of view (FOV) = 35 × 35 mm^2^) was acquired using the refocused echoes (RARE) sequence as a localization standard. Then Blood Oxygenation Level Dependent Magnetic Resonance Imaging (BOLD‐fMRI) imaging was performed using the EPI sequence with the set parameters; TR/TE = 1500/20 ms, layer thickness = 1 mm, matrix = 120 × 80, FOV = 24 × 24 mm^2^.

### 2.10 Imaging Data Analysis

In the fMRI data processing flow, the raw DICOM format data were first converted to NIFTI format and preliminary image quality assessment and correction were performed. Subsequently, the first volume of each subject was selected as the baseline, and the remaining volumes were spatially realigned using a six‐degree‐of‐freedom rigid‐body transformation algorithm to eliminate the effect of head movement and generate the average functional image. To minimize individual anatomical differences, all subject data were converted to standard template space by voxel‐level alignment. Next, a [3 3 3] FWHM Gaussian kernel was applied to spatially smooth the normalized data to improve the signal‐to‐noise ratio. In the time dimension, a 0.01–0.1 Hz bandpass filter was used to eliminate high‐frequency noise with low‐frequency drift. Finally, bilateral hippocampi and prefrontal cortices were localized according to the Paxinos‐Watson rat brain atlas, and the amplitude of low‐frequency fluctuations (ALFF) across the whole brain was calculated using DPABI software (Chinese Academy of Sciences, Beijing, China).

### 2.11 Extraction and Culture of Primary Microglia in Rat Hippocampus

Primary microglia were extracted and cultured as described in the literature with some modifications (Pesti et al. 2024). Specifically, hippocampi were isolated from 24 h neonatal SD rats, digested with 0.25% Trypsin–EDTA (1×, Gibco Co., USA) for 20 min, blown into a homogeneous tissue suspension by gentle suction, and centrifuged at 1000 r/min for 5 min. The cells were resuspended and inoculated into poly‐lysine (Solarbio Co, China) coated culture dishes and cultured at 37°C in a 5% CO_2_ cell culture incubator. The whole solution was changed on the first and third days, and half solution was changed every 3 days thereafter. After 2 weeks of mixed culture, cells were isolated by shaking the dishes at 200 rpm/min on a 37°C shaker to obtain purified microglia. The cell purification rate was determined by immunofluorescence staining using ionized calcium‐binding junction molecule 1 (IBA1) (1:300, Catalog Number: 10904‐1‐AP, Proteintech Group Inc., Wuhan, China) (Figure S2a).

### 2.12 Lentiviral Transfection

Primary microglia were inoculated in 6‐well plates at a density of 1 × 10^6^ cells per well and continuously transfected with LV‐RUVBL2, LV‐shRNA‐RUVBL2, or LV‐Scramble for 24 h. After 24 h, the culture medium was replaced with fresh DMEM and incubated for an additional 48 h. Western blot was used to validate the effect of gene disruption on RUVBL2 expression levels (Figure S2e,f).

### 2.13 Cell Chronic Hypoxia Modeling and Grouping

To simulate the chronic hypoxia and sevoflurane exposure treatment model in vivo, cells were transferred to an incubator with 3% O_2_, 92% N_2_, and 5% CO_2_ for 3 h as a hypoxic control group (Hypoxia). Sevoflurane was delivered into the incubator at a flow rate of 2 L/min. The concentration of sevoflurane in the incubator was detected using an anesthetic gas detector (Datex‐Ohmeda, UK) to maintain it at 3% for 3 h as the hypoxic anesthesia group (Hypoxia + Sevo). 48 h after the completion of lentiviral transfection, the LV‐RUVBL2, LV‐shRNA‐RUVBL2, and LV‐Scramble groups were similarly placed into the sevoflurane hypoxic incubator and treated with sevoflurane exposure for 3 h.

Primary microglia were divided into 5 groups to investigate the effect of RUVBL2 expression on the SGs formed by microglia when subjected to hypoxic sevoflurane injury (Table 2).

**TABLE 2:** Experimental grouping of primary hippocampal microglia.

Experimental grouping	Group name	Model establishment
Group 1	Hypoxia	3% O_2_, 92% N_2_, 5% CO_2_(3 h)
Group 2	Hypoxia+Sevo	Hypoxia+2 L/min sevoflurane (3 h)
Group 3	LV‐Scramble	Transfection with LV‐Scramble+ Hypoxia+2 L/min sevoflurane (3 h)
Group 4	RUVBL2‐KD	Transfection with LV‐shRNA‐RUVBL2 + Hypoxia+2 L/min sevoflurane (3 h)
Group 5	RUVBL2‐OE	Transfection with LV‐RUVBL2 + Hypoxia+2 L/min sevoflurane (3 h)

### 2.14 Western Blot Analysis

Rat hippocampal tissues and primary microglia were harvested, and total proteins were extracted using RIPA lysis buffer (no. P0013B, Beyotime, Shanghai, China) supplemented with a protease inhibitor cocktail (no. CW2200S, CoWin Biotech Co. Ltd., Jiangsu, China), phosphatase inhibitor cocktail (no. CW2383S, CoWin Biotech Co. Ltd., Jiangsu, China), and phenylmethane sulfonyl fluoride (PMSF) (Beyotime, Shanghai, China). The samples were lysed thoroughly on ice and then centrifuged at 4°C for 10 min. The supernatants were collected, and protein concentrations were determined using a BCA protein assay kit (no. P0012, Beyotime, Shanghai, China). Equal amounts of 15 μg protein per sample were separated by SDS‐PAGE electrophoresis and then transferred onto PVDF membranes. The membranes were incubated overnight at 4°C with primary antibodies: anti‐RUVBL2 (1:1000, Catalog Number: ab19027, Abcam, Cambridge, UK), anti‐α‐Ketoglutarate Dehydrogenase (OGDH) (1:1000, Catalog Number: 15212‐1‐AP, Proteintech Group Inc., Wuhan, China), anti‐Pyruvate Kinase 2 (PKM2) (1:1000, Catalog Number: 15822‐1‐AP, Proteintech Group Inc., Wuhan, China), anti‐CD86 (1:1000, Catalog Number: 13395‐1‐AP, Proteintech Group Inc., Wuhan, China), anti‐Arginase 1 (Arg1) (1:1000, Catalog Number: 16001‐1‐AP, Proteintech Group Inc., Wuhan, China), and anti‐Tubulin (1:50,000, Catalog Number: 10094‐1‐AP, Proteintech Group Inc., Wuhan, China) as the internal reference. After three washes with Tris‐buffered saline containing Tween‐20 (TBST), the membranes were incubated with the appropriate secondary antibodies at room temperature for 1 h. Following additional washing, the PVDF membranes were exposed to enhanced chemiluminescence (ECL) reagent (Elabscience Biotechnology Co. Ltd., Wuhan, China) for visualization and imaging. The gray values of the target bands were quantified using ImageJ (National Institutes of Health, Bethesda, USA). Relative protein expression levels were calculated by determining the ratio of the gray value of each target band to that of the Tubulin band.

### 2.15 Immunofluorescence

Rat brain tissue was fixed in 4% paraformaldehyde for 24 h, followed by sucrose gradient dehydration with each step lasting 24 h. Frozen sections of rat brain tissues, approximately 15 μm thick, were prepared. The sections and cells were permeabilized with 0.3% Triton X‐100, blocked with 5% goat serum (Invitrogen, R37624), and incubated overnight at 4°C with the following primary antibodies: anti‐IBA1 (1:300, Abcam, Catalog Number: ab283319, Cambridge, UK), anti‐GTPase‐activating protein (SH3 domain) binding protein 1 (G3BP1) (1:500, Cell Signaling Technology, Catalog Number: E8N8F, Danvers, MA, USA), anti‐IBA1 (1:200, Catalog Number: 10904‐1‐AP, Proteintech Group Inc., Wuhan, China), and anti‐G3BP1 (1:1000, Catalog Number: 66486–1‐Ig, Proteintech Group Inc., Wuhan, China). On the following day, the samples were rewarmed at 37°C for 1 h, washed three times with PBST, and incubated at 37°C for 1 h with fluorescent secondary antibodies: Alexa Fluor 488‐conjugated goat anti‐mouse (1:200, Catalog Number: ab150113, Abcam, Cambridge, UK), Alexa Fluor 594‐conjugated goat anti‐rabbit (1:200, Catalog Number: ab150080, Abcam, Cambridge, UK), Alexa Fluor 488‐conjugated goat anti‐rabbit (1:500, Catalog Number: ab150077, Abcam, Cambridge, UK), and Alexa Fluor 594‐conjugated goat anti‐mouse (1:300, Catalog Number: ab150116, Abcam, Cambridge, UK). Nuclei were stained with 4,6‐Diamidino‐2‐phenylindole dihydrochloride (DAPI) (Catalog Number: C0065, Solarbio, Beijing, China), followed by blocking with an anti‐fluorescence attenuation quencher. Fluorescence images were captured using a Zeiss LSM 900 laser confocal scanning microscope and a Leica STELLARIS laser confocal scanning microscope, both with a 40 × objective. Quantitative fluorescence analysis was performed using ImageJ (National Institutes of Health, Bethesda, USA).

### 2.16 Rat Blood Collection

Three days after ORIF surgery, blood collection was performed from the rat tail vein according to the standard method described in previous literature (Lee and Goosens 2015), ensuring procedural consistency, stable sample quality, and minimal stress to the animals. The rats were placed in a dedicated restrainer and maintained in a conscious state to avoid interference from anesthetics with blood parameters. The tail was soaked in 40°C–45°C warm water for 2–3 min to facilitate vasodilation. The blood collection area on the tail was then swabbed with 70% medical alcohol to expose the tail vein. A 23–25 G sterile needle was gently inserted along the lateral tail vein at a 20°–30° angle to the tail axis; after successful puncture, blood was collected slowly by drawing back the syringe plunger. The volume of blood collected per procedure was controlled at 0.5–0.6 mL. After blood collection, sterile cotton balls were applied gently to the puncture site for 30–60 s until hemostasis was achieved. The collected blood was transferred to centrifuge tubes and allowed to stand at room temperature for 30 min. Serum separation was then performed using a high‐speed refrigerated centrifuge at 3000 r/min and 4°C for 15 min. The upper serum layer was carefully aspirated and stored at −80°C for subsequent assays.

### 2.17 Pyruvate and Lactate Assays

According to the instructions, hippocampal tissue was taken and weighed (g), then nine times the volume (ml) of saline was added, mechanically homogenized, and centrifuged at 2500 r/min for 10 min, and the supernatant was taken, and then the protein concentration was determined by using the BCA, at which time the homogenized concentration of the protein was measured to be 10%. Pyruvate and lactate contents in rat hippocampal tissue, serum and supernatant of primary microglia were measured using a pyruvate assay kit (A081‐1‐1, Nanjing Jiancheng Bioengineering Institute, Wuhan, China) and a lactate assay kit (A019‐2‐1, Nanjing Jiancheng Bioengineering Institute, Wuhan, China). The lactate/pyruvate (L/P) ratio was calculated to assess the level of glycolytic metabolism.

### 2.18 ATP Level Measurement

The ATP content in the hippocampal tissue and primary hippocampal microglia of aged MCI rats was measured using the Enhanced ATP Assay Kit (no. S0027, Beyotime, Shanghai, China). According to the instructions, protein samples were prepared, and the supernatant was used to determine protein concentration using the BCA Protein Assay Kit (no. P0012, Beyotime, Shanghai, China). In a black opaque 96‐well plate, 20 μL of the protein sample homogenate and 100 μL of the ATP detection working solution were added and incubated at room temperature for 5 min. The luminescence was measured with a microplate luminometer (Victor Nivo 5F, Perkin Elmer Instruments Co. Ltd., MA, USA).

### 2.19 ATPase Activity Assay

To investigate the changes in ATPase activity in hippocampal tissue and primary hippocampal microglia of aged MCI rats after sevoflurane anesthesia and surgical trauma, the hippocampal tissue was prepared into a 10% protein homogenate according to the instructions and then diluted to 1% with physiological saline. The ATPase activity in the rat hippocampal tissue was measured using the ultra‐micro total ATPase assay kit (A070‐1, Jiancheng Bioengineering Institute, Nanjing, China).

### 2.20 Extracellular Acidification Rate and Oxygen Consumption Rate Assay

Primary hippocampal microglia were resuspended in complete DMEM medium and seeded at a density of 2 × 10^5^ cells/ml into black 96‐well microplates. The cells were incubated in a humidified environment at 37°C with 5% CO_2_. Working solutions for extracellular acidification rate (ECAR, E‐BC‐F069, Elabscience Biotechnology Co. Ltd., Wuhan, China) and oxygen consumption rate (OCR, E‐BC‐F068, Elabscience Biotechnology Co. Ltd., Wuhan, China) were freshly prepared. After treating the cells with 3% sevoflurane combined with hypoxia for 3 h, the cells were incubated with the ECAR and OCR assay working solutions at 37°C for 30 min in the dark. ECAR and OCR levels were subsequently measured using a multimode fluorescence microplate reader (Victor Nivo 5F, PerkinElmer Instruments Co. Ltd., Massachusetts, USA). The slopes of the fluorescence curves were used to quantify ECAR and OCR values.

### 2.21 Enzyme‐Linked Immunosorbent Assay (ELISA) Kit

Interleukin‐1β (IL‐1β) and interleukin‐10 (IL‐10) levels in hippocampal tissue homogenates and primary microglial culture supernatants were quantified using ELISA kits, following the manufacturer's instructions. The ELISA kits used were: IL‐1β (ER1094, 96T, FineTest Biotechnology Co. Ltd., Wuhan, China) and IL‐10 (ER0033, 96T, FineTest Biotechnology Co. Ltd., Wuhan, China). Optical density (OD) absorbance at 450 nm was measured using a microplate reader. The concentration of IL‐1β and IL‐10 in the samples was determined by constructing a standard curve, based on the proportional relationship between target analyte concentration and the OD450 value.

### 2.22 Image Acquisition and Analysis

MRI data were acquired using a Bruker 9.4T BioSpec 94/30 system (Bruker, Ettlingen, Germany) for multimodal scanning. T2‐weighted imaging (T2WI) was performed using a RARE sequence (TR/TE = 2500/33 ms, slice thickness = 1 mm, matrix = 256 × 256, FOV = 35 × 35 mm^2^) as the localization reference. This was followed by BOLD‐fMRI using an EPI sequence with parameters: TR/TE = 1500/20 ms, slice thickness = 1 mm, matrix = 120 × 80, FOV = 24 × 24 mm^2^. Bilateral hippocampi and prefrontal cortices were localized according to the Paxinos‐Watson rat brain atlas, and whole‐brain ALFF was calculated using DPABI software (Chinese Academy of Sciences, Beijing, China). Immunofluorescence images were captured using a 40 × objective on Zeiss LSM 900 and Leica STELLARIS laser confocal scanning microscopes. ImageJ software (National Institutes of Health, Bethesda, USA) was used to analyze fluorescence intensity, SG diameter, number, IBA1‐SGs colocalization, microglial morphology, branch number, as well as the quantification of gray values in Western blot assays, with Tubulin used as the internal reference.

### 2.23 Statistical Analysis

Statistical analysis was performed using GraphPad Prism software (version 10.1.2, San Diego, CA, USA). Use the Shapiro–Wilk test to assess the normality of the data distribution. A *t*‐test was used between two groups and a one‐way analysis of variance (ANOVA) was used between multiple groups. Tukey's post hoc test was performed for all experiments. All experiments were repeated at least three times, and *p* < 0.05 was considered statistically significant (*p < 0.05, ** *p* < 0.01, ***p < 0.001, and ****p < 0.0001).

## 3 Results

### 3.1 Sevoflurane Anesthesia and Surgery Is Associated With Postoperative Cognitive Dysfunction and Metabolic Reprogramming in Aged MCI Rats

**FIGURE 1:**
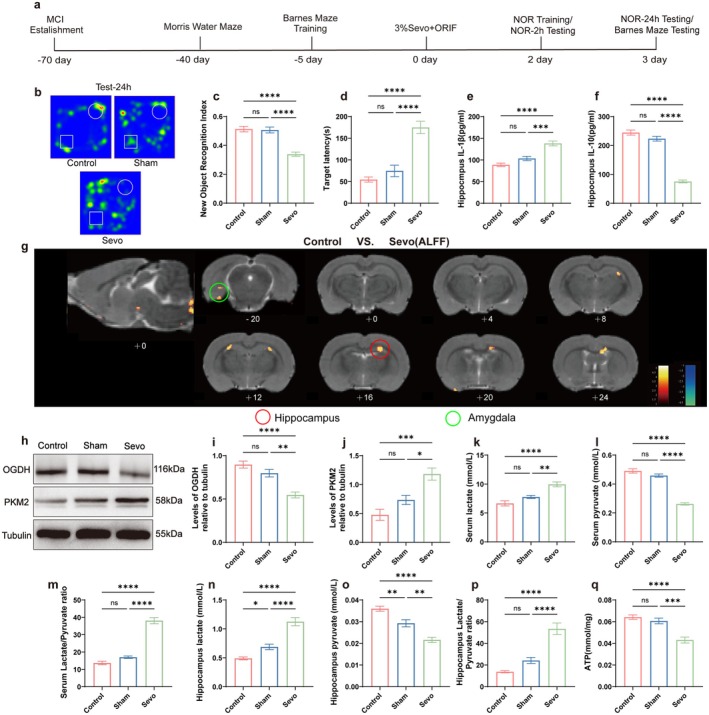
Sevoflurane anesthesia and surgery lead to postoperative cognitive dysfunction and metabolic reprogramming in aged MCI rats. (a) MCI and ORIF model establishment, behavioral assessment time chart. (b) NOR‐24 h represents the trajectory plot. (c) NOR‐24 h index (*n* = 10). (d) Barnes Maze testing period target latency (*n* = 10). (e) ELISA for hippocampal IL‐1β concentration (pg/mL) (*n* = 6). (f) Hippocampal anti‐inflammatory factor IL‐10 concentration (pg/mL) (*n* = 6). (g) Representative superimposed thermogram profiles of voxel‐wise analysis of brain ALFF values between Control and Sevo groups (*n* = 3, two‐sample *t*‐test, *p* < 0.01, cluster size > 50). Warm colors indicate brain regions with enhanced functional activity. (h) Western blot analysis of representative protein bands of OGDH and PKM2 in the hippocampus. (i) OGDH expression quantitative analysis (*n* = 6). (j) PKM2 expression quantitative analysis (*n* = 6). (k, l) Serum lactate and pyruvate levels (mmol/L) (*n* = 6). (m) Serum lactate/pyruvate ratio (L/P ratio) (*n* = 6). (n, o) Hippocampal lactate and pyruvate levels (mmol/L) (*n* = 6). (p) Hippocampal lactate/pyruvate ratio (L/P ratio) (*n* = 6). (q) ATP expression levels (mmol/mg) (*n* = 6). *p < 0.05, **p < 0.01, ***p < 0.001, and ****p < 0.0001, values are presented as the means ± SEMs.

The Barnes maze and NOR tests were conducted to assess the effects of 3‐h, 3% sevoflurane anesthesia during surgery on postoperative cognitive function in aged MCI rats (Figure 1a). No rats exhibited hypoxia or mortality during anesthesia, surgery, or behavioral testing. Analysis of the 2‐h and 24‐h NOR tests revealed that the NOR index in the Sevo group was significantly lower than in the Control and Sham groups (*p* < 0.05, Figure 1b,c, Figure S1c,d). In the Barnes maze test, the escape latency time in the Sevo group was notably longer than in the Control and Sham groups (*p* < 0.05, Figure 1d). Additionally, fMRI multimodal scans were performed 3 days after ORIF surgery. The ALFF technique measures spontaneous neural activity by analyzing the amplitude of low‐frequency BOLD signals (0.01–0.08 Hz) during the resting state (Tomasi and Volkow 2011), with elevated values indicating increased neuronal activity in specific brain regions. Compared to the Sevo group, the Control group showed a significant increase in ALFF within the hippocampal region and amygdala, suggesting functional activation of the hippocampus involved in learning, memory, and environmental adaptation (Figure 1g). ELISA results indicated that pro‐inflammatory cytokine IL‐1β expression was significantly elevated, while anti‐inflammatory cytokine IL‐10 expression was decreased in the Sevo group (*p* < 0.05, Figure 1e,f). These results demonstrate that sevoflurane anesthesia and surgery induce hippocampus‐dependent postoperative cognitive dysfunction and neuroinflammation in aged MCI rats.

To further investigate the mechanisms underlying POD in aged MCI rats and its relationship to glucose metabolism, Western blot analysis was performed to examine the expression levels of OGDH, a rate‐limiting enzyme in the tricarboxylic acid (TCA) cycle, and PKM2, a key enzyme in glycolysis, in the hippocampus. Additionally, a glycolysis product assay kit was used to measure lactate and pyruvate levels in hippocampal tissue and serum, and the L/P ratio was calculated to assess glycolytic activity. ATP levels were measured using an ATP assay kit to evaluate energy balance. Western blot results showed that, compared to the Control and Sham groups, the Sevo group exhibited decreased OGDH expression and increased PKM2 expression in the hippocampus (*p* < 0.05, Figure 1h–j). Furthermore, the Sevo group displayed significantly elevated lactate levels and reduced pyruvate levels in both hippocampal tissue and serum, along with a significantly increased L/P ratio (*p* < 0.05, Figure 1k–p), and reduced ATP levels (*p* < 0.05, Figure 1q). These results suggest that in aged MCI rats exposed to ORIF under sevoflurane anesthesia, metabolic reprogramming occurs, shifting from oxidative phosphorylation (OXPHOS) to glycolysis, with enhanced glycolytic activity and ATP energy deficiency.

### 3.2 Surgery Under Sevoflurane Anesthesia Elevated Hippocampal Microglial Pro‐Inflammatory Response Profiles in Aged MCI Rats

**FIGURE 2:**
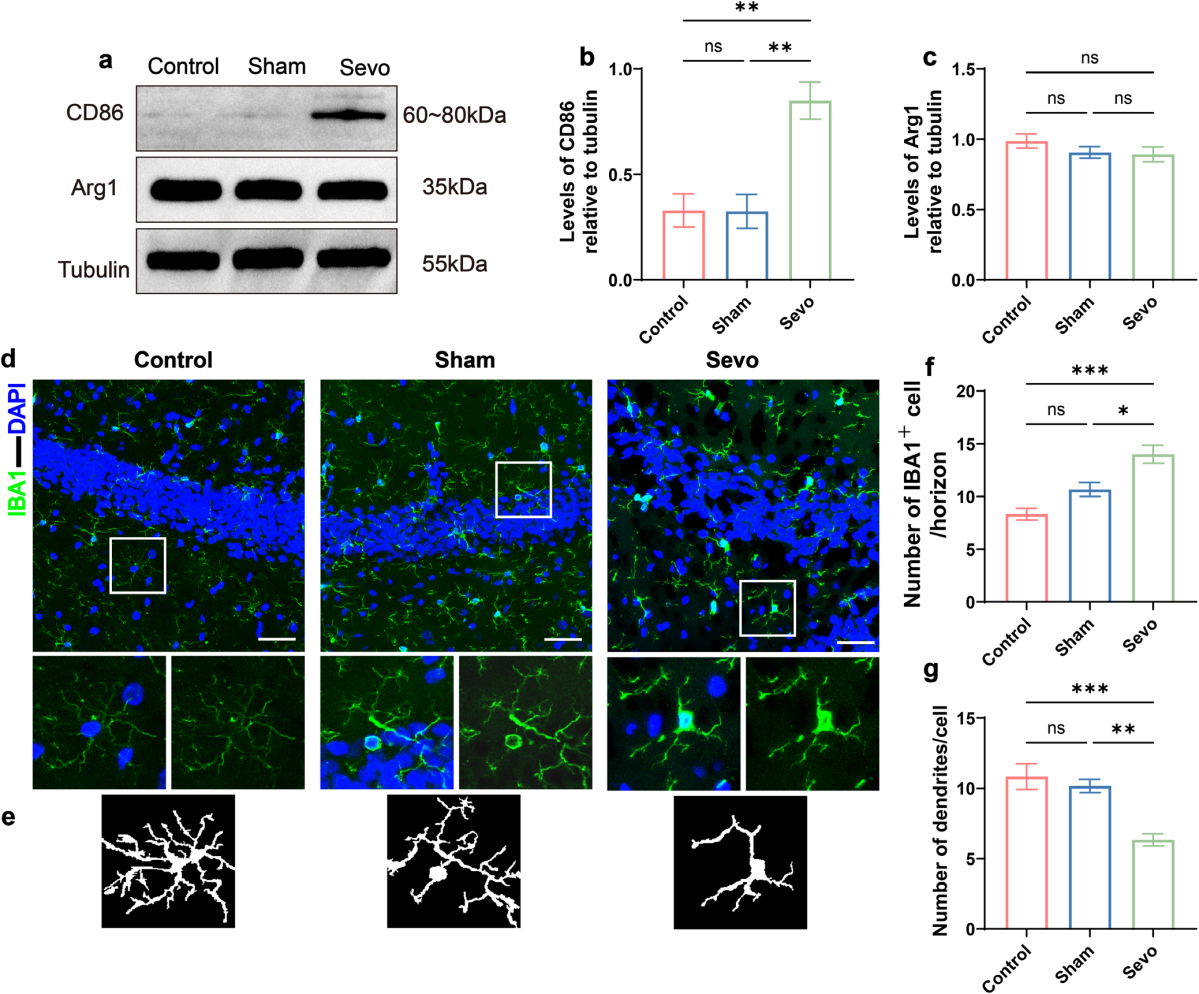
Surgery under sevoflurane anesthesia elevated hippocampal microglial pro‐inflammatory response profiles in aged MCI rats. (a) Western blot analysis of representative protein bands for CD86 and Arg1 in the hippocampus of aged MCI rats. (b) Quantitative analysis of CD86 expression (*n* = 6). (c) Quantitative analysis of Arg1 expression (*n* = 6). (d) Confocal microscopy images of IBA1 in the DG region of the hippocampus. Scale bar = 100 μm. (e) Morphological analysis of microglia. (f) Number of IBA1^+^ cells/horizon (*n* = 6). (g) Number of branches/cell (*n* = 6). *p < 0.05, **p < 0.01, ***p < 0.001, and ****p < 0.0001, values are presented as the means ± SEMs.

Postoperative cognitive dysfunction, induced by surgical trauma and anesthesia, is linked to abnormal microglial activation (Zeng et al. 2025; Zhang et al. 2016). To further elucidate the hippocampal microglial response to ORIF under sevoflurane anesthesia, Western blot analysis was performed to assess the expression levels of CD86 and Arg1, along with immunofluorescence staining to evaluate the IBA1 positivity rate in the dentate gyrus (DG) region of the hippocampus. Morphological analysis of microglia was also conducted. The results revealed that, in response to this combined challenge, hippocampal microglia in aged MCI rats exhibited a reactive state. This state was characterized by a significant increase in CD86 expression in the Sevo group (*p* < 0.05, Figure 2a,b), with no significant change in Arg1 expression compared to other groups (*p* > 0.05, Figure 2a,c). Additionally, the number of IBA1^+^ cells in the DG region of the hippocampus in the Sevo group was significantly higher (*p* < 0.05, Figure 2d,f), and morphological analysis revealed a significant reduction in the number of microglial branches (*p* < 0.05, Figure 2e,g). These results indicate that sevoflurane anesthesia combined with surgical trauma induces a shift in hippocampal microglial state toward a pro‐inflammatory profile, characterized by elevated CD86 expression and altered morphology.

### 3.3 Surgery Under Sevoflurane Anesthesia Increases SGs Aggregation and Endogenous Expression of RUVBL2

**FIGURE 3:**
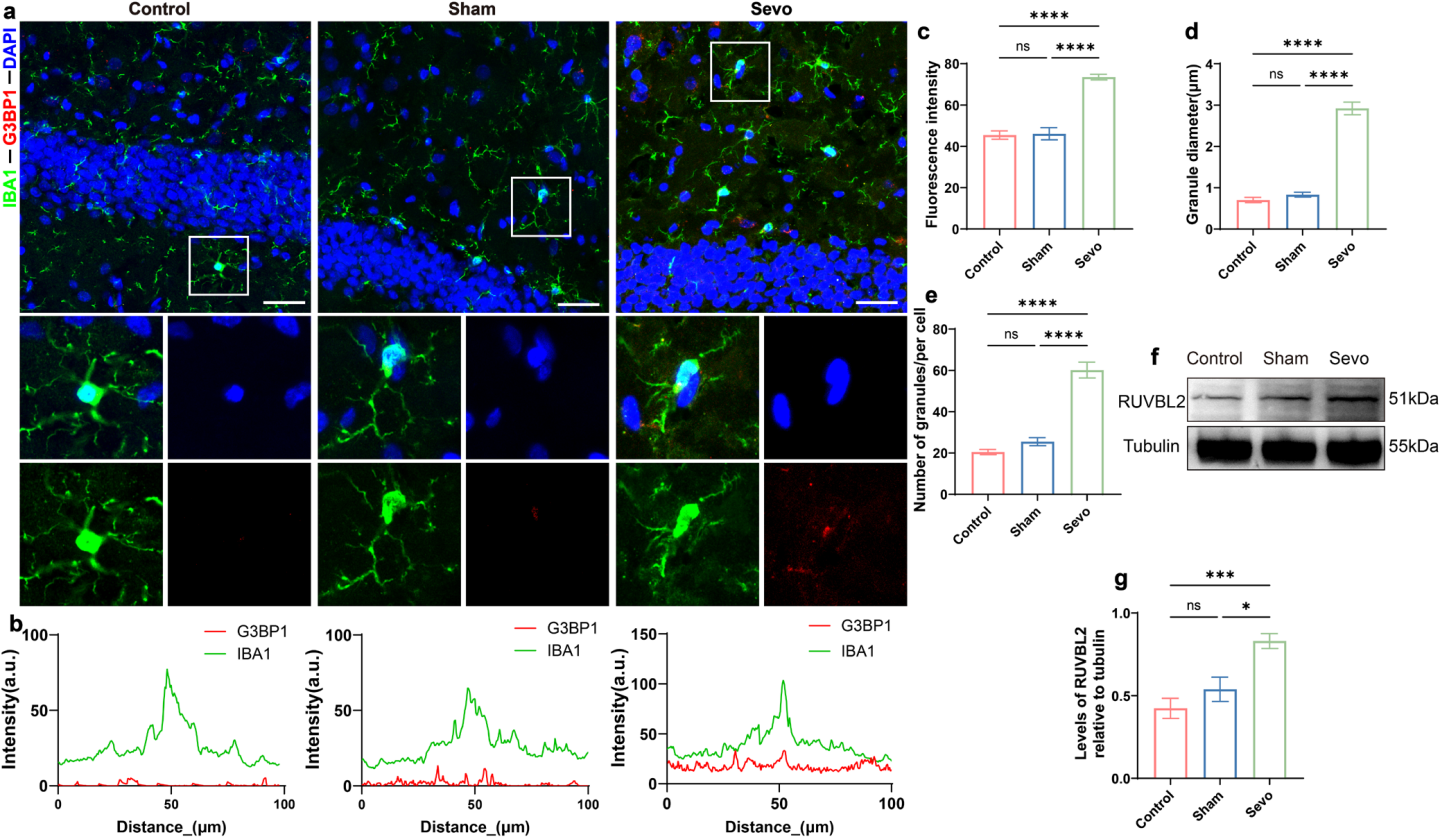
Surgery under sevoflurane anesthesia increases SGs aggregation and endogenous expression of RUVBL2. (a) Representative confocal microscopy images showing co‐expression of IBA1 and G3BP1 in the DG region of the hippocampus. Scale bar = 100 μm. (b) Fluorescence colocalization analysis of IBA1 and G3BP1 within the white rectangular box. (c) G3BP1 fluorescence intensity (*n* = 6). (d) SGs diameter (*n* = 6). (e) Number of SGs (*n* = 6). (f) Western blot analysis of RUVBL2 protein expression in the hippocampus of aged MCI rats. (g) Quantitative analysis of RUVBL2 protein expression (*n* = 6). *p < 0.05, **p < 0.01, ***p < 0.001, and ****p < 0.0001, values are presented as the means ± SEMs.

RUVBL2, a highly conserved ATPase belonging to the AAA^+^ protein family, has been identified as a core component of SGs, playing a critical role in their assembly dynamics and biological functions (Jain et al. 2016). Therefore, this study investigated the relationship between microglial SG aggregation and RUVBL2 expression in the context of sevoflurane anesthesia and surgery. Immunofluorescence analysis of IBA1‐SGs co‐localization revealed enhanced fluorescence intensity of G3BP1 in the hippocampal DG region in the Sevo group (*p* < 0.05, Figure 3c), a larger number of SGs with larger particle sizes in activated microglia (*p* < 0.05, Figure 3a,d,e), and increased co‐localization of IBA1‐SGs (*p* < 0.05, Figure 3b). Western blot analysis demonstrated that RUVBL2 expression was significantly higher in the Sevo group compared to the Control and Sham groups (*p* < 0.05, Figure 3f,g). These results indicate that sevoflurane anesthesia and ORIF surgery induce increased endogenous RUVBL2 expression and SG aggregation in the hippocampus of aged MCI rats.

### 3.4 Knockdown of RUVBL2 Ameliorates Sevoflurane Anesthesia and Surgery‐Induced Postoperative Cognitive Dysfunction in Aged MCI Rats

**FIGURE 4:**
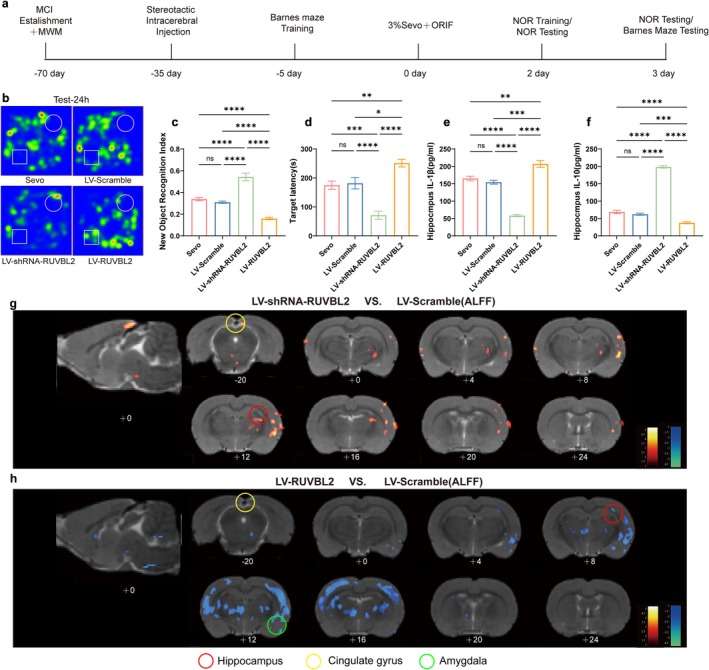
Knocking down RUVBL2 improves postoperative cognitive impairment in aged MCI rats. (a) Experimental workflow diagram. (b) NOR‐24 h represents the trajectory plot. (c) NOR‐24 h index (*n* = 10). (d) Barnes Maze escape latency (*n* = 10). (e) ELISA detection of hippocampal IL‐1β concentration (pg/ml) (*n* = 6). (f) Hippocampal IL‐10 concentration (pg/ml) (*n* = 6). (g) Representative superimposed thermogram profiles of voxel‐wise analysis of brain ALFF values between LV‐shRNA‐RUVBL2 and LV‐Scramble groups (*n* = 3, two‐sample *t*‐test, *p* < 0.01, cluster size > 50). (h) Representative superimposed thermogram profiles of voxel‐wise analysis of brain ALFF values between LV‐RUVBL2 and LV‐Scramble groups (*n* = 3, two‐sample *t*‐test, *p* < 0.01, cluster size > 50). Warm colors indicate brain regions with enhanced functional activity. *p < 0.05, **p < 0.01, ***p < 0.001, and ****p < 0.0001, values are presented as the means ± SEMs.

Our previous experimental data indicate that sevoflurane anesthesia and surgical trauma induce POD in aged MCI rats. To further investigate whether RUVBL2 expression influences cognitive function, this study employed stereotactic brain injection to deliver lentiviruses targeting RUVBL2 into the hippocampus of elderly MCI rats, thereby modulating gene expression. Injection of LV‐RUVBL2 enhanced RUVBL2 expression, while LV‐shRNA‐RUVBL2 reduced its expression. To rule out the potential effects of the viral vector on gene expression, an empty vector (LV‐Scramble) was also used as a control. Behavioral analysis revealed no significant differences in the NOR‐2 h index, NOR‐24 h index and Barnes maze escape latency between the Sevo and LV‐Scramble groups (*p* > 0.05, Figure 4b–d, Figure S1h,i), suggesting that the viral vector and the stereotactic injection procedure had minimal impact on cognitive function in aged MCI rats. Thus, observed effects can be attributed to the specific functions of the target gene. Compared to the Sevo and LV‐Scramble groups, the LV‐shRNA‐RUVBL2 group exhibited significantly increased NOR‐2 h index, NOR‐24 h index and reduced Barnes maze escape latency (*p* < 0.05, Figure 4b–d, Figure S1h,i). Conversely, the LV‐RUVBL2 group showed decreased NOR‐2 h index, NOR‐24 h index, and increased escape latency in the Barnes maze (*p* < 0.05, Figure 4b–d, Figure S1h,i). ALFF analysis revealed significantly enhanced neural activity in the hippocampal region, cingulate gyrus, and amygdala in the LV‐shRNA‐RUVBL2 group (Figure 4g). In contrast, the LV‐RUVBL2 group displayed reduced signaling in these areas (Figure 4h), indicating that RUVBL2 overexpression impairs spontaneous neural activity and hippocampal function. Furthermore, ELISA results showed that IL‐1β expression was significantly decreased in the LV‐shRNA‐RUVBL2 group, while IL‐10 levels were increased. In contrast, the LV‐RUVBL2 group exhibited elevated pro‐inflammatory cytokine levels and reduced anti‐inflammatory cytokine expression. No significant differences in inflammatory factor expression were observed between the LV‐Scramble and Sevo groups (*p* > 0.05, Figure 4e,f). These results demonstrate that knockdown of RUVBL2 alleviates hippocampus‐dependent postoperative cognitive and memory impairments, as well as neuroinflammation, in aged MCI rats exposed to sevoflurane anesthesia and surgery.

### 3.5 Knockdown of RUVBL2 Attenuates Sevoflurane Anesthesia and Surgery‐Induced Postoperative Cognitive Dysfunction in Aged MCI Rats by Inhibiting Metabolic Reprogramming

**FIGURE 5:**
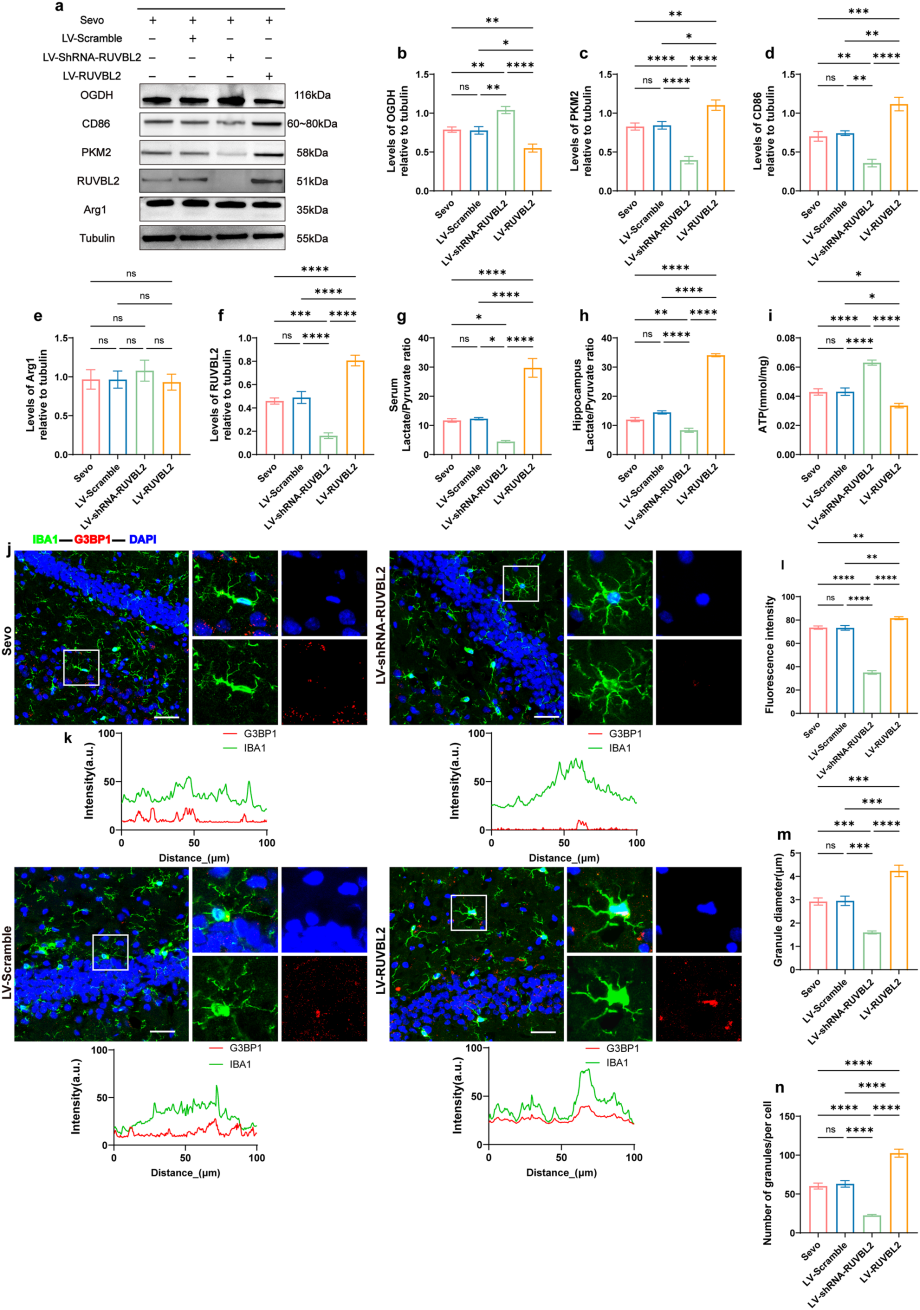
Knocking down RUVBL2 alleviates POD in aged MCI rats induced by sevoflurane anesthesia and surgery by inhibiting metabolic reprogramming. (a) Western blot detection of hippocampal OGDH, PKM2, RUVBL2, CD86, and Arg1 representative protein blots. (b) OGDH expression quantitative analysis (*n* = 6). (c) PKM2 expression quantitative analysis (*n* = 6). (d) Quantitative analysis of CD86 expression (*n* = 6). (e) Quantitative analysis of Arg1 expression (*n* = 6). (f) RUVBL2 expression quantitative analysis (*n* = 6). (g) Serum lactate/pyruvate ratio (L/P ratio) (*n* = 6). (h) Hippocampal lactate/pyruvate ratio (L/P ratio) (*n* = 6). (i) ATP expression levels (mmol/mg) (*n* = 6). (j) Representative confocal microscopy images of IBA1 and G3BP1 co‐expression in the hippocampus. Scale bar = 100 μm. (k) Fluorescence colocalization analysis of IBA1 and G3BP1 within the white rectangular box. (l) G3BP1 fluorescence intensity (*n* = 6). (m) SGs diameter (*n* = 6). (n) Number of SGs (*n* = 6). *p < 0.05, **p < 0.01, ***p < 0.001, and ****p < 0.0001, values are presented as the means ± SEMs.

Further validation was conducted to determine whether regulating RUVBL2 expression affects metabolic reprogramming and, in turn, influences microglial response and SG recruitment. The metabolic characteristics and IBA1‐SGs colocalization were assessed in rats from the Sevo, LV‐Scramble, LV‐shRNA‐RUVBL2, and LV‐RUVBL2 groups. Compared to the Sevo and LV‐Scramble groups, the LV‐RUVBL2 group exhibited downregulated OGDH expression and upregulated PKM2 expression (*p* < 0.05, Figure 5a–c). Additionally, lactate levels and the L/P ratio in hippocampal tissue and serum were significantly elevated, while ATP levels were reduced (*p* < 0.05, Figure 5g–i, Figure S1k–n). In contrast, the LV‐shRNA‐RUVBL2 group attenuated the increased glycolytic activity in the brains of aged MCI rats following sevoflurane anesthesia and ORIF. These results indicate that RUVBL2 knockdown prevents metabolic reprogramming induced by sevoflurane anesthesia and surgical trauma, halting the increase in glycolysis and restoring ATP levels. Moreover, Western blot results revealed a downregulation of CD86 expression in the LV‐shRNA‐RUVBL2 group, while CD86 expression was upregulated in the LV‐RUVBL2 group compared to the Sevo and LV‐Scramble groups (*p* < 0.05, Figure 5d). No significant difference in Arg1 expression was observed across the groups (*p* > 0.05, Figure 5e). Immunofluorescence analysis showed that the LV‐shRNA‐RUVBL2 group exhibited the fewest SGs, which were the smallest in size, whereas the LV‐RUVBL2 group had a higher number of SGs with larger particle sizes, enhanced IBA1‐SGs colocalization, and increased G3BP1 fluorescence intensity (*p* < 0.05, Figure 5j–n). In conclusion, these data suggest that reduced RUVBL2 expression inhibits metabolic reprogramming progression and effectively alleviates postoperative cognitive deficits in aged MCI rats subjected to sevoflurane anesthesia and surgical trauma.

### 3.6 Sevoflurane Induces Metabolic Reprogramming and SGs Aggregation in Hypoxic Primary Hippocampal Microglia

**FIGURE 6:**
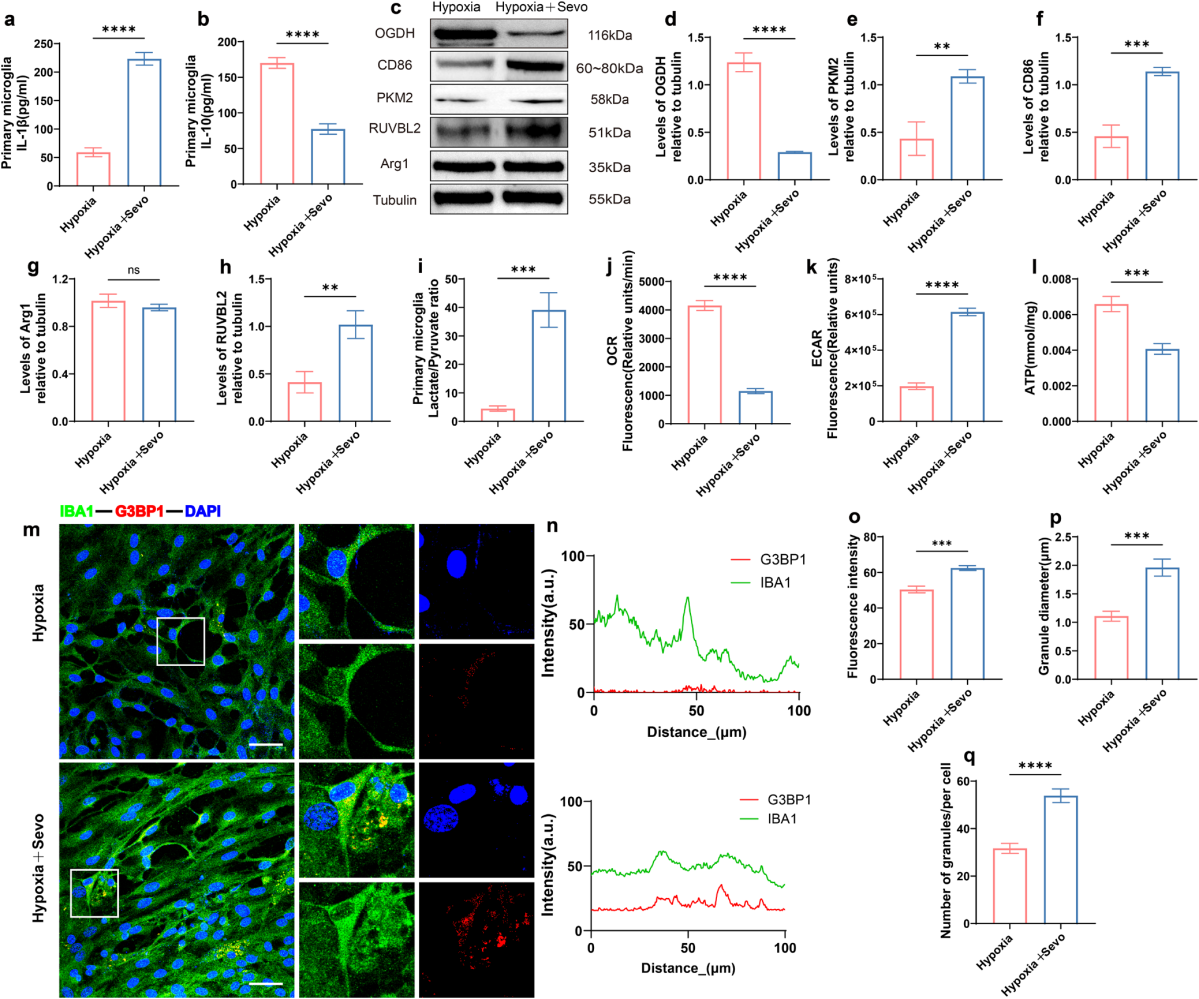
Sevoflurane induces proinflammatory factor expression and metabolic reprogramming in primary hippocampal microglia. (a) ELISA detection of IL‐1β concentration (pg/mL) in cell supernatant (*n* = 6). (b) IL‐10 concentration in cell culture supernatant (pg/mL) (*n* = 6). (c) Western blot detection of representative protein blots of OGDH, CD86, PKM2, RUVBL2 and Arg1 in primary hippocampal microglia from rats. (d) Quantitative analysis of OGDH expression (*n* = 6). (e) Quantitative analysis of PKM2 expression (*n* = 6). (f) Quantitative analysis of CD86 expression (*n* = 6). (g) Quantitative analysis of Arg1 expression (*n* = 6). (h) Quantitative analysis of RUVBL2 expression (*n* = 6). (i) Lactate/pyruvate ratio (L/P ratio) in cell culture supernatant (*n* = 6). (j) OCR relative fluorescence (*n* = 6). (k) ECAR relative fluorescence (*n* = 6). (l) ATP expression level (mmol/mg) (*n* = 6). (m) Representative confocal microscopy images of co‐expression of IBA1 and G3BP1 in primary hippocampal microglia. Scale bar = 100 μm. (n) Fluorescence colocalization analysis of IBA1 and G3BP1 within the white rectangular box. (o) G3BP1 fluorescence intensity (*n* = 6). (p) SGs diameter. (*n* = 6). (q) Number of SGs. (*n* = 6). *p < 0.05, **p < 0.01, ***p < 0.001, and ****p < 0.0001, values are presented as the means ± SEMs.

To further investigate whether glucose metabolism changes in microglia contribute to metabolic reprogramming, primary microglia were isolated and cultured from the hippocampus of 24‐h‐old newborn SD rats. In vitro, sevoflurane exposure was simulated by treating cells with hypoxia for 3 h under conditions of 3% O_2_, 92% N_2_, and 5% CO_2_ (Hypoxia group), and with 3% sevoflurane combined with hypoxia for 3 h (Hypoxia + Sevo group). Microglia are crucial in regulating neuroinflammation (Wang, Chen, et al. 2025; Zhai et al. 2022; Zhang et al. 2020). ELISA results showed increased IL‐1β expression in the Hypoxia + Sevo group, indicating heightened pro‐inflammatory responses, while IL‐10 expression was reduced (*p* < 0.05, Figure 6a,b). Compared to the Hypoxia group, the Hypoxia + Sevo group exhibited downregulated OGDH expression and upregulated PKM2 expression (*p* < 0.05, Figure 6c–e). Lactate levels and the L/P ratio in the cell supernatant were significantly elevated (*p* < 0.05, Figure 6i,S2b). Furthermore, OCR and ECAR levels were measured, with OCR reflecting cellular oxygen consumption related to OXPHOS and ECAR indicating glycolytic activity (Dai et al. 2024). Compared to the Hypoxia group, the Hypoxia + Sevo group showed a decrease in OCR (*p* < 0.05, Figure 6j), an increase in ECAR (*p* < 0.05, Figure 6k), and a decrease in ATP levels (*p* < 0.05, Figure 6l). These results suggest that sevoflurane treatment shifts the metabolic mode of hypoxic cells from OXPHOS to enhanced glycolysis, accompanied by a reduction in ATP levels. Combined with in vivo findings, this suggests that changes in microglial metabolic patterns are a key factor in metabolic reprogramming in aged MCI rats, characterized by a shift from OXPHOS to glycolysis in microglial glucose metabolism.

Metabolic reprogramming is a hallmark of immune cell polarization (Mak et al. 2017; Makowski et al. 2020). Therefore, the effects of sevoflurane on the activation characteristics of primary microglia under hypoxic conditions were further examined. Results showed that, compared to the Hypoxia group, CD86 expression was significantly increased in the Hypoxia + Sevo group (*p* < 0.05, Figure 6f), while no significant change was observed in Arg1 expression (*p* > 0.05, Figure 6g). After sevoflurane exposure, RUVBL2 expression in hypoxic primary hippocampal microglia was significantly upregulated (*p* < 0.05, Figure 6h). Fluorescence analysis demonstrated an increase in the number and size of SGs in primary microglia, enhanced co‐localization of IBA1 and SGs, and elevated fluorescence intensity of G3BP1 (*p* < 0.05, Figure 6m–q). These results confirm that sevoflurane exposure induces an increase in RUVBL2 expression in hypoxic primary microglia, triggers pro‐inflammatory responses, and leads to enhanced SG accumulation and elevated inflammatory expression.

### 3.7 Knockdown of RUVBL2 Attenuates Sevoflurane‐Induced Pro‐Inflammatory Response Profiles and SGs Aggregation in Hypoxic Primary Hippocampal Microglia by Inhibiting Metabolic Reprogramming

**FIGURE 7:**
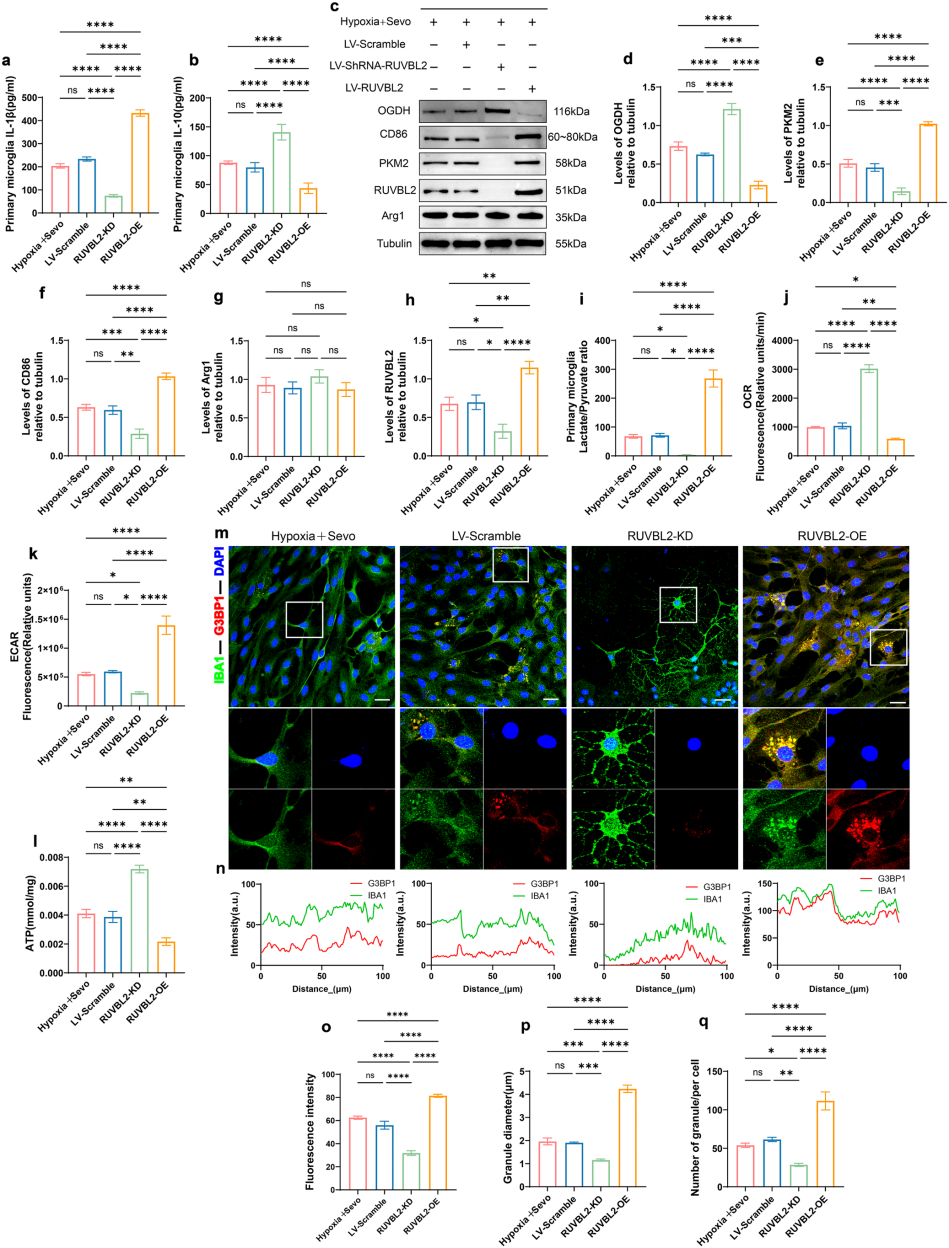
Knockdown of RUVBL2 attenuates sevoflurane‐induced pro‐inflammatory response profiles and SGs aggregation in hypoxic primary hippocampal microglia by inhibiting metabolic reprogramming. (a) ELISA detection of IL‐1β concentration (pg/mL) (*n* = 6). (b) IL‐10 concentration (pg/mL) (*n* = 6). (c) Western blot detection of representative protein blots of OGDH, PKM2, CD86, Arg1, and RUVBL2 in hippocampal primary microglia. (d) Quantitative analysis of OGDH expression (*n* = 6). (e) Quantitative analysis of PKM2 expression (*n* = 6). (f) Quantitative analysis of CD86 expression (*n* = 6). (g) Quantitative analysis of Arg1 expression (*n* = 6). (h) Quantitative analysis of RUVBL2 expression (*n* = 6). (i) Lactate/pyruvate ratio (L/P ratio) in cell culture supernatant (*n* = 6). (j) Relative fluorescence of OCR (*n* = 6). (k) Relative fluorescence of ECAR (*n* = 6). (l) ATP expression levels (mmol/mg) (*n* = 6). (m) Representative confocal microscopy images of co‐expression of IBA1 and G3BP1 in rat hippocampal primary microglia. Scale bar = 100 μm. (n) Fluorescence colocalization analysis of IBA1 and G3BP1 within the white rectangular box. (o) G3BP1 fluorescence intensity (*n* = 6). (p) SGs diameter (*n* = 6). (q) Number of SGs (*n* = 6). *p < 0.05, **p < 0.01, ***p < 0.001, and ****p < 0.0001, values are presented as the means ± SEMs.

Primary microglia transfected with LV‐Scramble, LV‐shRNA‐RUVBL2, and LV‐RUVBL2 were exposed to a hypoxic environment with 3% sevoflurane for 3 h, designated as the LV‐Scramble, RUVBL2‐KD, and RUVBL2‐OE groups, respectively. Compared to the Hypoxia + Sevo and LV‐Scramble groups, the RUVBL2‐KD group exhibited reduced IL‐1β expression and attenuated IL‐10 expression, while the RUVBL2‐OE group showed an opposite trend (*p* < 0.05, Figure 7a,b). The RUVBL2‐KD group displayed lower glycolytic activity, with decreased PKM2 expression and increased OGDH expression (*p* < 0.05, Figure 7c–e), a significant reduction in the L/P ratio in the cell supernatant (*p* < 0.05, Figure 7i), enhanced OCR (*p* < 0.05, Figure 7j), decreased ECAR (*p* < 0.05, Figure 7k), and increased ATP levels (*p* < 0.05, Figure 7l). In contrast, the RUVBL2‐OE group exhibited heightened glycolytic activity, suggesting that RUVBL2 knockdown mitigates the glycolytic enhancement induced by hypoxia and sevoflurane. Furthermore, CD86 expression, associated with pro‐inflammatory signaling, was significantly reduced in the RUVBL2‐KD group compared to the Hypoxia + Sevo and LV‐Scramble groups (*p* < 0.05, Figure 7f), while RUVBL2 overexpression resulted in increased CD86 expression (*p* < 0.05, Figure 7h). No significant differences in Arg1 expression were observed across the groups (*p* > 0.05, Figure 7g). Immunofluorescence analysis revealed that in the RUVBL2‐KD group, primary hippocampal microglia exhibited smaller and fewer SGs, reduced IBA1‐SG co‐localization, and decreased fluorescence intensity of G3BP1 (*p* < 0.05, Figure 7m–q). In conclusion, these results demonstrate that RUVBL2 plays a pivotal role in regulating metabolic reprogramming in microglia. By modulating glucose metabolism, RUVBL2 attenuates the shift to a reactive microglial state characterized by pro‐inflammatory signaling, reducing SG accumulation, and mitigating neuroinflammation, thereby maintaining neuronal homeostasis.

## 4 Discussion

This study aimed to elucidate the underlying mechanisms linking RUVBL2‐regulated microglial metabolic reprogramming to POD in aged MCI rats. Experimental findings confirmed that sevoflurane anesthesia combined with surgical stress induces microglial metabolic reprogramming, initiating a dynamic pathological cascade characterized by metabolic disturbances, heightened microglial responsiveness to inflammatory signals, and abnormal SG aggregation, all of which contribute to the development of POD. Targeted knockdown of RUVBL2 effectively disrupted this pathological process, restoring microglial metabolic balance, inhibiting excessive inflammatory responses, and preventing SG accumulation, ultimately leading to significant alleviation of POD in aged MCI rats.

Delirium is widely recognized as an acute form of brain failure, reflecting a reduced homeostatic reserve in vulnerable individuals (Inouye et al. 2014; Saczynski et al. 2014; Slooter et al. 2020). POD, defined as delirium occurring within 1 week post‐anesthesia and surgery, typically peaks between Days 1 and 3 (Devinney et al. 2018). Several cohort studies have shown that individuals who experience POD are more likely to develop long‐term cognitive deficits, including postoperative cognitive dysfunction and accelerated AD progression, compared to those who do not develop POD (Demanet et al. 2024; Huang et al. 2021; Inouye et al. 2016; Lingehall et al. 2017). Additionally, a lower preoperative cognitive status has been identified as an independent risk factor for postoperative cognitive dysfunction (Chen et al. 2022; Saczynski et al. 2012). In this study, an aged MCI rat model combined with an ORIF model was employed to simulate clinical POD scenarios in elderly populations. Behavioral analysis revealed that in the Sevo group, rats exhibited a decreased NOR index and prolonged Barnes maze escape latency, indicating that sevoflurane anesthesia combined with surgical trauma impairs hippocampus‐dependent memory and induces POD. Notably, RUVBL2 knockdown mitigated these adverse outcomes.

Microglia rapidly respond to subtle pathological changes in the central nervous system (CNS), clearing cellular debris and phagocytosing protein aggregates to promote tissue repair (Pan et al. 2025; van Olst et al. 2025). Upon abnormal activation, microglial metabolism undergoes a characteristic shift, resembling the Warburg effect observed in tumor cells, transitioning from OXPHOS to glycolysis, accompanied by a reduction in ATP production (Lauro and Limatola 2020). Growing evidence indicates that microglial metabolic reprogramming plays a critical role in their activation (Cheng et al. 2021; Fumagalli et al. 2018), particularly in neuroinflammation and perioperative neurocognitive disorders (PND) (Xiao et al. 2018; Zhu, Ji, et al. 2020). Glucose is converted into pyruvate and lactate, with their extracellular concentrations serving as indicators of glycolytic activity. The lactate‐to‐pyruvate (L/P) ratio reflects the extent to which pyruvate enters the TCA cycle for aerobic metabolism or is converted into lactate through anaerobic metabolism, highlighting mitochondrial dysfunction. A higher L/P ratio suggests a greater reliance on glycolysis and enhanced glycolytic activity (Jalloh et al. 2015; Luo et al. 2021). Multivariate logistic regression analyses have shown that lactate and the L/P ratio are positive predictors of mortality, whereas pyruvate is a negative predictor (Taylor et al. 2022; Timofeev et al. 2011). In M1‐activated macrophages, glycolysis is the dominant metabolic pathway, leading to increased lactate accumulation (Xie et al. 2024; Ye et al. 2022). In this study, elevated lactate levels, decreased pyruvate levels, and an increased L/P ratio were observed in the hippocampal tissue and serum of the Sevo group, indicating enhanced glycolytic activity. Targeting and modulating neuroglial glucose metabolism thus presents a promising strategy for mitigating neuroinflammation and the associated neurocognitive deficits (Han et al. 2021; Procaccini, Santopaolo, et al. 2016; Wang, Liu, et al. 2024).

Laura C. Graham and colleagues identified OGDH as a key regulator of synaptic vulnerability across brain regions when analyzing synaptic vulnerability characteristics in non‐human primate brains (Graham et al. 2019). A study investigating mRNA‐miRNA networks in the prefrontal cortex and hippocampus of patients with AD, as well as AD mouse models, highlighted OGDH as a core gene with significantly reduced expression, suggesting its potential as a novel biomarker for AD (Huang et al. 2022). In lipopolysaccharide (LPS)‐induced macrophages, OGDH expression was significantly downregulated, indicating a disruption of the TCA cycle (Zhao et al. 2022). Proteomics studies further confirmed that pro‐inflammatory immune cells exhibit high activity of key glycolytic enzymes, and their proliferation is dependent on glycolytic activity (Procaccini, Carbone, et al. 2016). Knockdown of PKM2 inhibits glycolysis (Ying et al. 2021), whereas high PKM2 expression and metabolic activity enhance glycolytic metabolism and promote immune cell activation toward a pro‐inflammatory phenotype (Wynn et al. 2013; Zhai et al. 2023; Zhu, Ji, et al. 2020), thereby regulating neuroinflammation and cognitive impairments in PND (Wang, Shen, et al. 2024). Additionally, PKM2 influences microglia–neuron/astrocyte interactions, maintaining metabolic homeostasis, energy supply, and neuroprotective immune responses (Lee et al. 2022). In the present study, the Sevo group exhibited increased IBA1^+^ microglia numbers and significantly upregulated CD86 expression in the hippocampus, accompanied by elevated PKM2 expression and reduced OGDH expression, indicating enhanced pro‐inflammatory responses. Consistent expression patterns were observed in vitro. Further metabolic assays revealed that the Hypoxia + Sevo group exhibited elevated ECAR, indicative of hyperactivated glycolytic activity, and decreased OCR, suggesting impaired mitochondrial respiration (Ghosh et al. 2018). These findings demonstrate that sevoflurane anesthesia and surgery can induce metabolic reprogramming in microglia, promoting a shift toward a reactive state characterized by a pro‐inflammatory functional profile. This study reveals a close spatiotemporal link between microglial metabolic reprogramming, cellular state transitions with enhanced inflammatory signaling, neuroinflammation, and the development of POD. Furthermore, the research confirms that elevated postoperative cerebral lactate levels and an increased L/P ratio are closely associated with POD, highlighting the pivotal role of immunometabolism in POD plasticity. These insights offer new perspectives for future investigations of POD from a metabolic perspective.

SGs are dynamic, membrane‐bound cellular organelles formed through liquid–liquid phase separation (LLPS) under various stress conditions. They consist of untranslated mRNAs, RNA‐binding proteins such as T‐cell intracellular antigen 1 (TIA1), G3BP1/2, and translation initiation factors (Kedersha et al. 2013; Zhou et al. 2024). The disassembly or inhibition of SG aggregation and accumulation can halt the progression of neurodegenerative diseases (Alberti et al. 2019; Sato et al. 2023; Yuan et al. 2025), a process that is ATP‐dependent (Begovich and Wilhelm 2020; Cereghetti et al. 2021). RUVBL2, a core ATPase subunit of the R2TP complex (RUVBL1‐RUVBL2‐Tah1‐Pih1), regulates the transition between the open and closed states of the RUVBL1/RUVBL2 hexameric ring through contraction of its ATP‐binding pocket, thus maintaining the complex's role in regulating cell metabolism and protein homeostasis (Dauden et al. 2021). Additionally, RUVBL2 regulates the mTOR signaling pathway via its ATPase activity, influencing glucose metabolism patterns. In a specific RUVBL2 knockout mouse model, reduced mTOR signaling activity alleviated insulin resistance‐related pathological phenotypes, including impaired glucose tolerance, hyperglycemia, and hyperlipidemia (Javary et al. 2018). ATP depletion induced by the inhibition of glycolysis with 2‐deoxyglucose (2‐DG) or the blockade of OXPHOS with carbonyl cyanide m‐chlorophenylhydrazone (CCCP) slows or halts the movement and fusion of SGs, leading to the formation of persistent SGs (Jain et al. 2016; Zaarur et al. 2015). This study focuses on how RUVBL2 regulates glucose metabolism and affects SGs. Anesthetic surgical stimulation upregulates the endogenous expression of RUVBL2 in aged POD rats and hippocampal primary microglia, triggering metabolic reprogramming and significant ATP reduction. Additionally, microglia exhibit clustering of more and larger SGs. In contrast, RUVBL2 knockdown ameliorates the inhibition of OXPHOS and SG kinetics. Microglia are particularly susceptible to SG formation, which is characterized by large SGs in the brains of elderly patients with late‐onset AD (Ghosh and Geahlen 2015; Wu et al. 2023). Impaired phagocytosis and activation of pro‐inflammatory microglia contribute to Aβ plaque accumulation, enhanced SG formation, and the release of harmful neuronal mediators, leading to neuroinflammation and neuronal damage (Thanos and Lukens 2025). In macrophages, knocking down RUVBL1 or RUVBL2 inhibits LPS‐induced pro‐inflammatory responses and decreases the expression of pro‐inflammatory genes such as nitric oxide synthase 2 (Nos2), IL‐1β, and nitric oxide (NO), thus regulating pro‐inflammatory signaling (Zhang et al. 2021). Experimental data demonstrate that RUVBL2 primarily affects SG assembly dynamics by regulating the shift in cellular glucose metabolism, which in turn modulates immune cell activation. These findings offer new insights into the relationship between SGs and the progression of PND at the level of energy metabolism. Collectively, the AAA^+^ ATPase RUVBL2 maintains systemic glucose, lipid, and acid–base homeostasis, highlighting its potential as a therapeutic target for metabolic syndrome.

## 5 Limitations of the Study

This study has several limitations. While it is the first to highlight the critical role of microglial metabolic reprogramming in POD in aged MCI rats and identifies RUVBL2 as a key regulatory target, offering new insights into the pathological mechanisms of POD, the metabolite dynamics in aged MCI rats under both physiological and pathological conditions warrant further investigation through metabolomics to elucidate additional regulatory nodes within the glucose metabolic pathway. Moreover, recent studies have demonstrated that dynamic shifts in the spatial heterogeneity of microglia significantly impact disease progression (Ardura‐Fabregat et al. 2025). Although challenges such as unstandardized nomenclature and overlapping markers remain, these novel state characterizations offer valuable insight into the continuous spectrum of microglial biology (Paolicelli et al. 2022). In this context, the present study focuses on the pro‐inflammatory response signature of microglia as a central aspect of their postoperative state, with the observed microglial responses serving as a key component of the broader metabolic‐inflamatory transition induced by anesthesia and surgery. To overcome the limitations of current characterization, future research will employ histological integration and dynamic tracking techniques to conduct more detailed analyses of the high heterogeneity of postoperative microglia, aiming to identify more precise intermediate functional phenotypes.

## 6 Conclusions

In conclusion, this study reveals a strong spatiotemporal link between microglial metabolic reprogramming and the pathogenesis of POD. Experimental findings demonstrate that RUVBL2 regulates the pro‐inflammatory response profile and SGs aggregation in hippocampal microglia by modulating their metabolic reprogramming, thereby influencing POD progression. Additionally, this study highlights the pivotal role of glucose metabolism in microglia across neurodegenerative diseases. Collectively, RUVBL2 emerges as a promising therapeutic target and novel intervention strategy for mitigating POD progression through the modulation of metabolic reprogramming.

## Author Contributions

Lin Zhang and Zixuan Wang performed molecular detection and fMRI scanning. Lin Zhang, Zixuan Wang, and Chenyi Yang drafted the manuscript. Chenyi Yang and Xinyi Wang conducted statistical analysis. Xing Liu, Haonan Zhang, and Huan Liu performed animal experiments. Huihui Liao and Jun Chen conducted morphological analysis. Haiyun Wang designed the study and secured funding. All authors read and approved the final manuscript. Lin Zhang, Zixuan Wang, and Chenyi Yang have contributed equally to this work.

## Funding

This work was supported by grants from The National Natural Science Foundation of China (82371205), Tianjin Health Research Project (TJWJ2023XK019), Tianjin Public Health Science and Technology Major Project (24ZXGZSY00180), and Tianjin Health Research Project (TJWJ2023QN042). We confirm all the funding information is accurate and compliant.

## Ethics Statement

All experimental protocols received approval from the Institutional Animal Care and Use Committee of Nankai Animal Resource Center, Nankai University (approval number: 2023‐SYDWLL‐000623).

## Consent

The authors have nothing to report.

## Conflicts of Interest

The authors declare no conflicts of interest.

## Supporting information


**Figure S1:** Screening of rat MCI model, behavioral assessment and detection of energy metabolism‐related indicators. (a) Representative test‐track plots of the MWM of rats in Control and MCI groups. (b) Escaping latency of MWM in Control and MCI rats (two‐sample *t*‐test). (c) NOR‐2 h represents the trajectory plot. (d) NOR‐2 h index (*n* = 10). (e) Hippocampal ATPase activity assay (U/mgprot) (*n* = 6). (f) Western blotting assay of representative protein blots for hippocampal RUVBL2 lentivirus transfection. (g) Quantitative analysis of RUVBL2 expression (*n* = 3). (h) NOR‐2 h represents the trajectory plot. (i) NOR‐2 h index (*n* = 10). (j) Hippocampal ATPase Activity assay (U/mgprot) (*n* = 6). (k) Rat serum lactate content (mmol/L) (*n* = 6). l Serum pyruvate content (mmol/L) (*n* = 6). (m) Lactate content of rat hippocampal tissue (mmol/L) (*n* = 6). (n) Pyruvate content of rat hippocampal tissue (mmol/L) (*n* = 6). *p < 0.05, **p < 0.01, ***p < 0.001, and ****p < 0.0001; values are presented as the means ± SEMs.

**Figure S2:** Identification of primary microglia in the hippocampus, lactate, pyruvate and ATPase activity assays. (a) IBA1 identification of primary microglia in the hippocampus. Scale bar = 100 μm. (b) Lactate content of the cell supernatant from hippocampal primary microglia (mmol/L) (*n* = 6). (c) Pyruvate content of the cell supernatant from hippocampal primary microglia (mmol/L) (*n* = 6). (d) ATPase Activity assay of hippocampal primary microglia (U/mgprot) (*n* = 6). (e) Western blot analysis of representative protein blots from RUVBL2 lentiviral transfections of hippocampal primary microglia. (f) Quantitative analysis of RUVBL2 expression (*n* = 3). (g) ATPase activity assay of hippocampal primary microglia (U/mgprot) (*n* = 6). (h) Lactate content of the cell supernatant from hippocampal primary microglia (mmol/L) (*n* = 6). (i) Pyruvate content of the cell supernatant from hippocampal primary microglia (mmol/L) (*n* = 6). *p < 0.05, **p < 0.01, ***p < 0.001, and ****p < 0.0001; values are presented as the means ± SEMs.

## Data Availability

The datasets used and/or analyzed during the current study are available from the corresponding author on reasonable request.
