# Supplementary figures and images for: RUVBL2 Regulates Microglia Metabolic Reprogramming to Mediate Stress Granules Aggregation Exacerbating Postoperative Delirium in Aged Mild Cognitive Impairment Rats
Source: Aging Cell. 2026 Apr 3;25(4):e70458. doi: 10.1111/acel.70458 (PMC13052178; doi:10.1111/acel.70458)

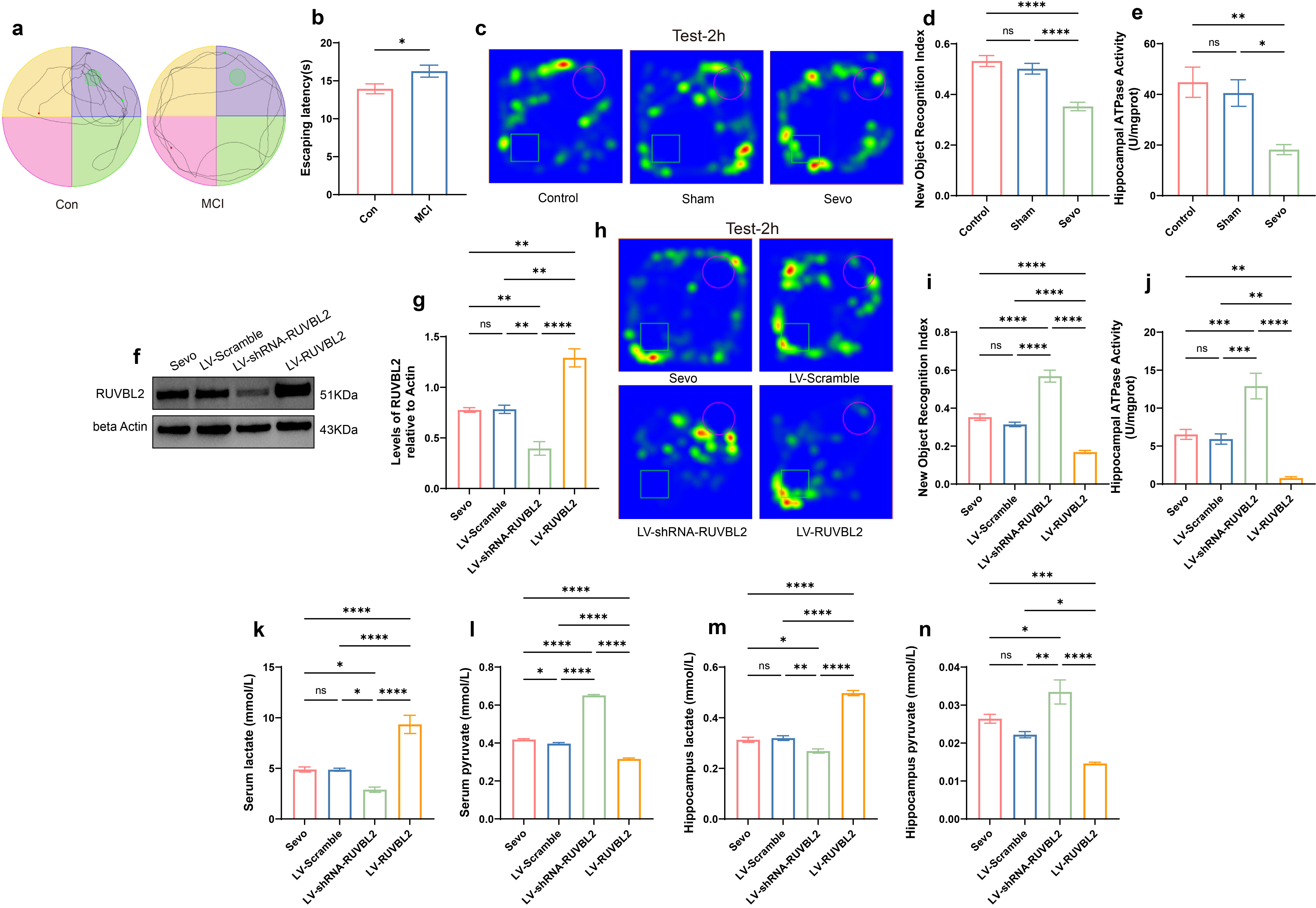

Supplement: Supplementary file 1 — Figure S1: Screening of rat MCI model, behavioral assessment and detection of energy metabolism‐related indicators. (a) Representative test‐track plots of the MWM of rats in Control and MCI groups. (b) Escaping latency of MWM in Control and MCI rats (two‐sample t‐test). (c) NOR‐2 h represents the trajectory plot. (d) NOR‐2 h index (n = 10). (e) Hippocampal ATPase activity assay (U/mgprot) (n = 6). (f) Western blotting assay of representative protein blots for hippocampal RUVBL2 lentivirus transfection. (g) Quantitative analysis of RUVBL2 expression (n = 3). (h) NOR‐2 h represents the trajectory plot. (i) NOR‐2 h index (n = 10). (j) Hippocampal ATPase Activity assay (U/mgprot) (n = 6). (k) Rat serum lactate content (mmol/L) (n = 6). l Serum pyruvate content (mmol/L) (n = 6). (m) Lactate content of rat hippocampal tissue (mmol/L) (n = 6). (n) Pyruvate content of rat hippocampal tissue (mmol/L) (n = 6). *p < 0.05, **p < 0.01, ***p < 0.001, and ****p < 0.0001; values are presented as the means ± SEMs. [file ACEL-25-e70458-s001.png]

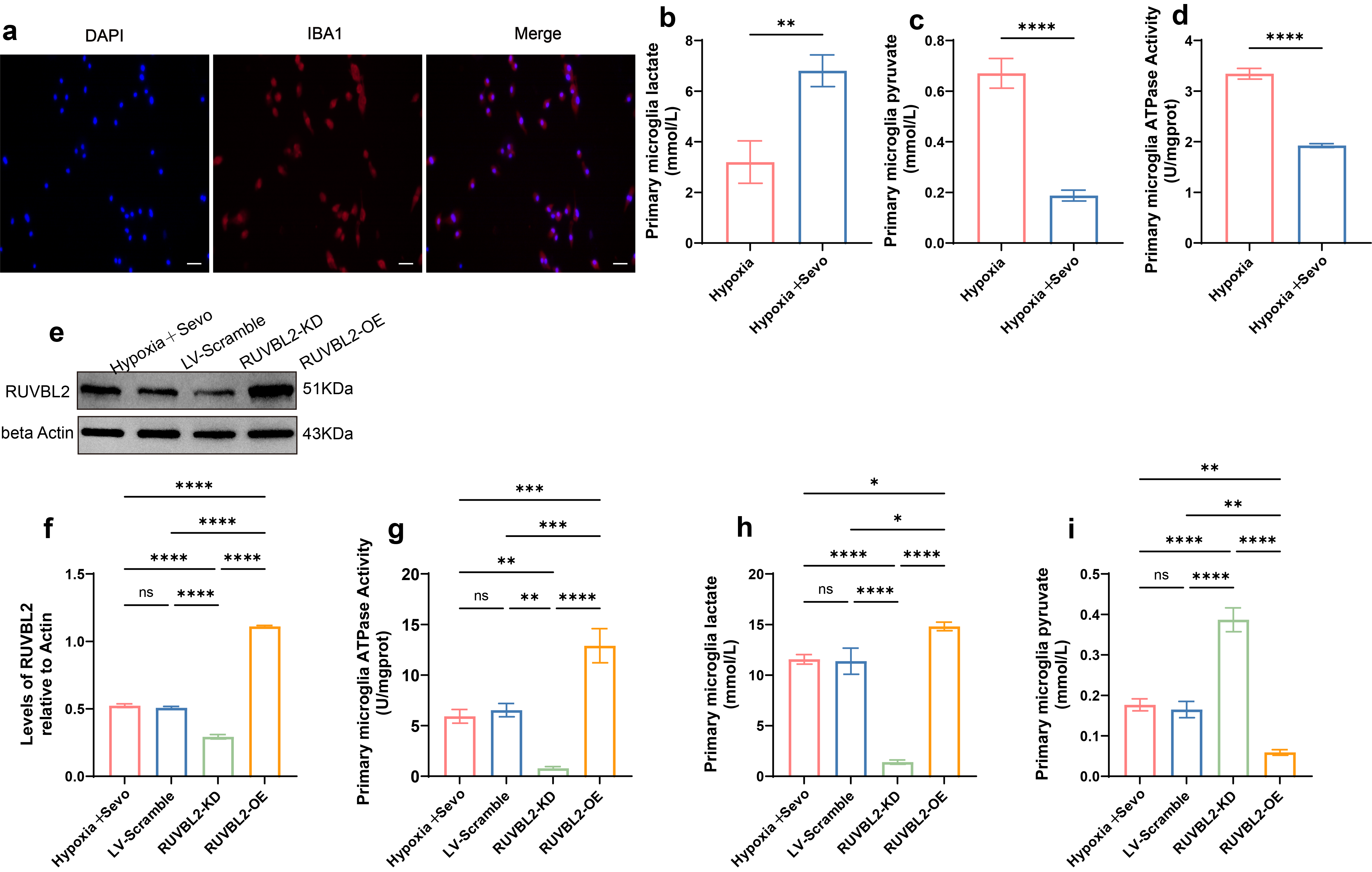

Supplement: Supplementary file 2 — Figure S2: Identification of primary microglia in the hippocampus, lactate, pyruvate and ATPase activity assays. (a) IBA1 identification of primary microglia in the hippocampus. Scale bar = 100 μm. (b) Lactate content of the cell supernatant from hippocampal primary microglia (mmol/L) (n = 6). (c) Pyruvate content of the cell supernatant from hippocampal primary microglia (mmol/L) (n = 6). (d) ATPase Activity assay of hippocampal primary microglia (U/mgprot) (n = 6). (e) Western blot analysis of representative protein blots from RUVBL2 lentiviral transfections of hippocampal primary microglia. (f) Quantitative analysis of RUVBL2 expression (n = 3). (g) ATPase activity assay of hippocampal primary microglia (U/mgprot) (n = 6). (h) Lactate content of the cell supernatant from hippocampal primary microglia (mmol/L) (n = 6). (i) Pyruvate content of the cell supernatant from hippocampal primary microglia (mmol/L) (n = 6). *p < 0.05, **p < 0.01, ***p < 0.001, and ****p < 0.0001; values are presented as the means ± SEMs. [file ACEL-25-e70458-s002.png]
